# Catalytic properties of trivalent rare-earth oxides with intrinsic surface oxygen vacancy

**DOI:** 10.1038/s41467-024-49981-9

**Published:** 2024-07-09

**Authors:** Kai Xu, Jin-Cheng Liu, Wei-Wei Wang, Lu-Lu Zhou, Chao Ma, Xuze Guan, Feng Ryan Wang, Jun Li, Chun-Jiang Jia, Chun-Hua Yan

**Affiliations:** 1 Key Laboratory for Colloid and Interface Chemistry, Key Laboratory of Special Aggregated Materials, School of Chemistry and Chemical Engineering, Shandong University, Jinan, 250100 China; 2 Department of Chemistry and Engineering Research Center of Advanced Rare-Earth Materials of Ministry of Education, Tsinghua University, Beijing, 100084 China; 3 Center for Rare Earth and Inorganic Functional Materials, School of Materials Science and Engineering & National Institute for Advanced Materials, Nankai University, Tianjin, 300350 China; 4 College of Materials Science and Engineering, Hunan University, Changsha, 410082 China; 5 Department of Chemical Engineering, University College London, Roberts Building, Torrington Place, London, WC1E 7JE UK; 6 Fundamental Science Center of Rare Earths, Ganjiang Innovation Academy, Chinese Academy of Sciences, Ganzhou, 341000 China; 7 Beijing National Laboratory for Molecular Sciences, State Key Lab of Rare Earth Materials Chemistry and Applications, PKU-HKU Joint Lab in Rare Earth Materials and Bioinorganic Chemistry, Peking University, Beijing, 100871 China

**Keywords:** Heterogeneous catalysis, Computational chemistry, Catalytic mechanisms

## Abstract

Oxygen vacancy (O_v_) is an anionic defect widely existed in metal oxide lattice, as exemplified by CeO_2_, TiO_2_, and ZnO. As O_v_ can modify the band structure of solid, it improves the physicochemical properties such as the semiconducting performance and catalytic behaviours. We report here a new type of O_v_ as an intrinsic part of a perfect crystalline surface. Such non-defect O_v_ stems from the irregular hexagonal sawtooth-shaped structure in the (111) plane of trivalent rare earth oxides (RE_2_O_3_). The materials with such intrinsic O_v_ structure exhibit excellent performance in ammonia decomposition reaction with surface Ru active sites. Extremely high H_2_ formation rate has been achieved at ~1 wt% of Ru loading over Sm_2_O_3_, Y_2_O_3_ and Gd_2_O_3_ surface, which is 1.5–20 times higher than reported values in the literature. The discovery of intrinsic O_v_ suggests great potentials of applying RE oxides in heterogeneous catalysis and surface chemistry.

## Abstract (web summary)

Oxygen vacancies (O_v_) are prevalent anionic defects in transition metal oxides, known for enhancing catalytic performance by modifying the material’s electronic and geometric structure. Here, the authors introduce a novel type of O_v_, and materials featuring this rare O_v_ structure demonstrate excellent performance in the ammonia decomposition reaction.

## Introduction

Oxygen vacancy (O_v_)^1–3^ is a ubiquitous anionic point defect in transition and *f*-element metal oxides. Commonly, the defect type of O_v_ requires cations with changeable multiple valence states such as Ce^3+/4+^ ^4–6^ and Ti^3+/4+^ ^7,8^. This defect is realized during the treatment in high temperature^9^ or reductive conditions^8,10^. The lattice or surface O^2−^ are taken away together with the reduction of metal cations while keeping the crystal structure, leaving O_v_ within the lattice. On the one hand, the role of O_v_ in heterogeneous catalysis has been widely reported^4–8^. For example, the generation of O_v_ in CeO_2_ (cubic fluorite structure, ${Fm}\bar{3}m$ space group) accompanied by the reduction of Ce^4+^ to Ce^3+^ changes its surface charge distribution and creates electrophilic sites^11^, which plays a crucial role in improving catalytic performance in CO oxidation, water-gas shift and CO_2_ reduction^5,6,9,10^. On the other hand, such redox process is hardly possible with irreducible oxides such as ZrO_2_, SiO_2_ and Al_2_O_3_, thereby preventing the generation of defect-based O_v_^12–15^.

In this work, we discover a new type of surface O_v_ that does not require a point defect formation nor the redox of metal cations. Such intrinsic O_v_ stems from the natural atomic arrangements in certain crystalline surface of rare earth oxide (RE_2_O_3_). We have analysed the structure and surface charge distribution of RE_2_O_3_ (such as Sm_2_O_3_, Y_2_O_3_ and Gd_2_O_3_) with body-centred cubic structure (*Ia3* space group) based on density functional theory (DFT). An irregular hexagonal sawtooth-shaped structure is found in the (111) surface of those RE_2_O_3_, forming intrinsic surface O_v_. Next to the O_v_ are penta-coordinated RE^3+^ with strong electrophilic nature. These RE_2_O_3_ with intrinsic surface O_v_ are loaded with Ru clusters as active metal (Ru/RE_2_O_3_, RE = Y, Sm and Gd) for ammonia decomposition reaction, in which an optimal N-binding strength is required. These Ru/RE_2_O_3_ catalysts exhibit excellent catalytic performance that is comparable to the most active Ru/CeO_2_ catalyst that is equipped with the defect O_v_^16^, despite that the RE cations are not redox active. During the reaction, the intrinsic O_v_ has desired absorption strength of NH_3_ at the neighbouring RE^3+^, and causes Ru species more reducible, which facilitates the initial N–H breaking. Such intrinsic O_v_ shows promise of utilizing their novel properties of RE_2_O_3_ in catalysis, providing suitable adsorption of reaction molecules for oxidation and hydrogenation chemistry.

## Results

### Intrinsic O_v_ in the RE_2_O_3_ surface

**Fig. 1:**
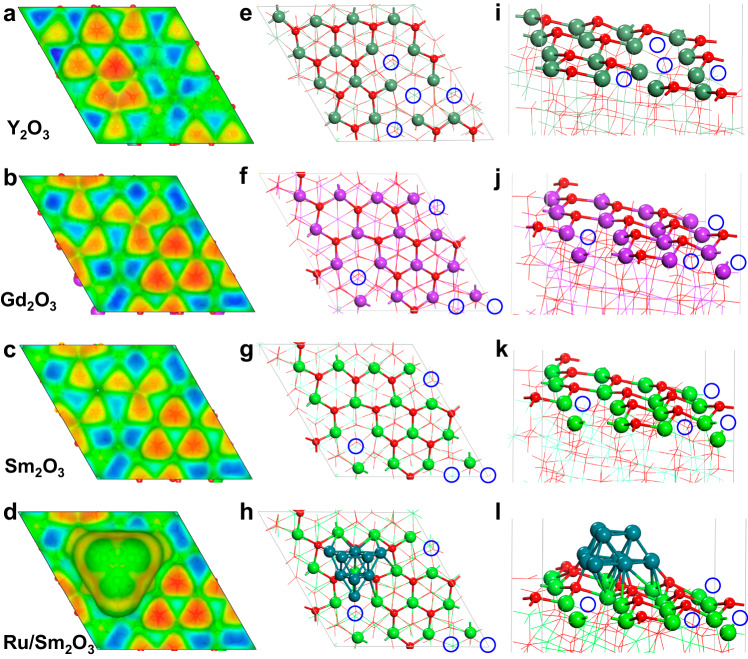
Structure and electrostatic potential results of DFT calculations simulating rare earth oxide (111) surfaces. **a**−**d** Electron density isosurface mapped with electrostatic potential surface of Y_2_O_3_(111), Gd_2_O_3_(111), Sm_2_O_3_(111), and Ru_9_/Sm_2_O_3_(111), respectively, at 0.003 e·bohr^−3^; **e**–**h** top views, and **i**−**l** side views of optimized surface structure of Y_2_O_3_(111), Gd_2_O_3_(111), Sm_2_O_3_(111), and Ru_9_/Sm_2_O_3_(111), respectively. Blue circles indicate oxygen vacancy or intrinsic surface O_v_ structures; blue and orange areas on electrostatic potential surface indicate electrophilic and nucleophilic sites, respectively.

Rare earth oxides with cubic structure, such as Sm_2_O_3_, Y_2_O_3_, and Gd_2_O_3_ mainly expose (111) surface at high temperatures or under harsh reaction conditions^5,17^. We found irregular hexagonal sawtooth-shaped structures formed by three 5-coordinated RE atoms and three 4-coordinated O atoms in cubic-phase Y_2_O_3_(111), Gd_2_O_3_(111) and Sm_2_O_3_(111) (Fig. 1 a−c, e−g, i−k). These vacancy structures are slightly different from the surface point defect O_v_ in CeO_2_(111), which is surrounded by three 6-coordinated Ce (Supplementary Fig. 1). Three RE-O bonds are broken for each oxygen vacancy, and thus 25% outmost oxygen vacancy are missing on the (111) surface. Similar O_v_ is also observed in Sm_2_O_3_(110) and (100) surfaces (Supplementary Fig. 2). Due to the exposure of unsaturated 5-coordinated RE atoms, these O_v_ are electrophilic. Such electrophilic sites can adsorb and activate electron-rich molecules, such as NH_3_ and H_2_O. The adsorption strength needs to be high enough to form a stable RE-N/O bond and not too high to cause surface poisoning. We have calculated the adsorption energy values of NH_3_ molecules on a series of RE_2_O_3_ and compared with the standard γ-Al_2_O_3_(111) surface (Supplementary Figs. 3−5 and Supplementary Table 1). The adsorption of NH_3_ on the Sm_2_O_3_(111) surface (−0.44 eV) was stronger than the Sm_2_O_3_(110) surface (−0.36 eV), but weaker than that on the Sm_2_O_3_(100) surface (−0.98 eV). The moderate adsorption of NH_3_ on Sm_2_O_3_(111) surface (−0.44 eV) is more favourable to the activation of NH_3_ molecule than that on γ-Al_2_O_3_(111) surface, because the latter exhibits an excessively strong adsorption of NH_3_ (−1.74 eV). Besides, in order to illustrate the unique role of intrinsic O_v_, which is the special spatial structure in catalysts, the adsorption of molecules at different Sm ions on the surface of Sm_2_O_3_ is investigated. The adsorption on the vacancy-related Sm sites is stronger than on the non-vacancy Sm sites of Sm_2_O_3_(111) surface (Supplementary Fig. 6 and Supplementary Table 1), where adsorption might be too weak for effective catalysis. Thus, intrinsic O_v_ on the Sm_2_O_3_(111) surface showed great superiority in the adsorption of molecules. Ru is a highly efficient active metal to catalyse the ammonia decomposition reaction^18^, so we have constructed a supported catalyst model of Ru_9_ clusters on Sm_2_O_3_(111) surface (Fig. 1d, h, l). The NH_3_ adsorption energy on Ru_9_/Sm_2_O_3_(111) is −0.79 eV, indicating that the addition of Ru promotes the adsorption of NH_3_ without causing strong binding in Ru_9_/Al_2_O_3_(111) (−1.88 eV). Therefore, RE_2_O_3_ support metal catalysts can be promising in catalysing reactions involving electron-rich molecules.

### Structure and catalytic performance of the Ru/RE_2_O_3_ catalysts

**Fig. 2:**
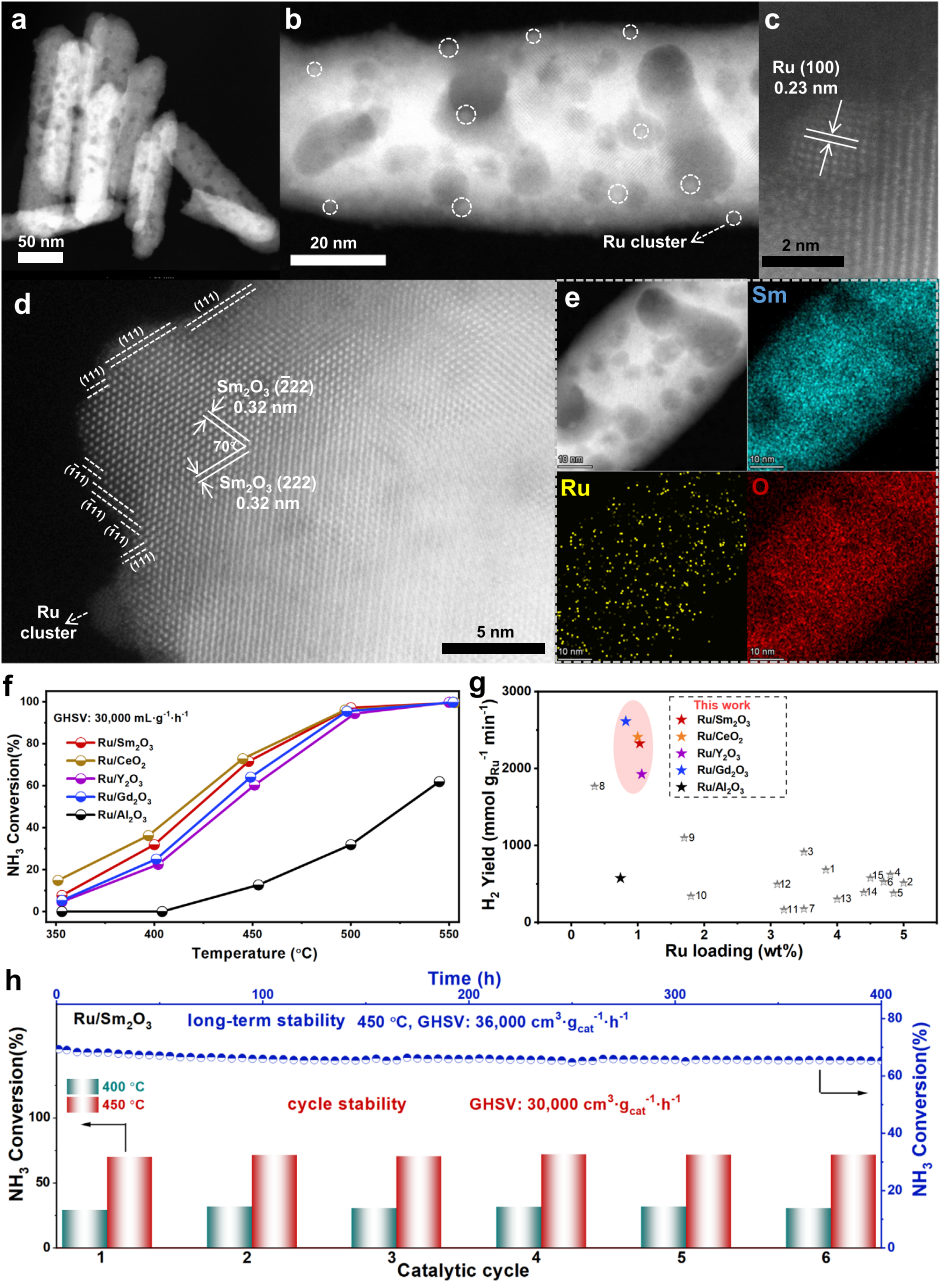
The aberration-corrected HAADF-STEM images and catalytic performance test of catalysts. **a**–**d** The aberration-corrected HAADF-STEM images of the used Ru/Sm_2_O_3_; **e** EDS elemental mapping results of the used Ru/Sm_2_O_3_; **f** temperature-dependent activities of the catalysts (Ru/Sm_2_O_3_, Ru/Y_2_O_3_, Ru/Gd_2_O_3_ and Ru/Al_2_O_3_, GHSV = 30,000 mL·g^−1^·h^−1^); **g** comparison of H_2_ yield with other Ru-based catalysts: 1: Ru/Sm_2_O_3_-p^20^, 2:Ru/Y_2_O_3_-p^21^, 3: K-Ru/MgO^22^, 4: K-Ru/CNTs^23^, 5: Ru/MgO-CNTs^24^, 6: Ru/c-MgO^25^, 7: Ru/CaAlO_*x*_-w^26^, 8: Ru-Ni/CeO_2_^27^, 9: Ru/MgO^28^, 10: Ru/LaCeO_*x*_^29^, 11: Ru/CNF^30^, 12: Ru-Cs-Mg/MIL-101^31^, 13: Ru/Al_2_O_3_^32^, 14: Ru-K/ZrO_2_^18^, 15: Ru-K/CNT^18^; (h) the long-term stability (GHSV = 36,000 mL·g^−1^·h^−1^) and the cycle stability test (GHSV = 30,000 mL·g^−1^·h^−1^) of Ru/Sm_2_O_3_ catalyst.

In order to explore the potential applications of the intrinsic surface O_v_ in RE_2_O_3_, a series of Ru/RE_2_O_3_ (Ru/Sm_2_O_3_, Ru/Y_2_O_3_, Ru/Gd_2_O_3_) and reference Ru/Al_2_O_3_ catalysts (~1 wt% Ru content, determined by ICP-MS, Supplementary Table 2) were prepared by colloidal deposition method. The transmission electron microscopy (TEM) images of the catalysts were shown in Supplementary Fig. 7. Ru/Sm_2_O_3_ and Ru/Gd_2_O_3_ had nanorods structure, while Ru/Y_2_O_3_ and Ru/Al_2_O_3_ had nanosheets and nanoparticles morphology, respectively. The high-angle annular dark-field scanning transmission electron microscopy (HAADF-STEM) images showed that the edge of Sm_2_O_3_ (Fig. 2d and Supplementary Fig. 8), Gd_2_O_3_ (Supplementary Fig. 9) and Y_2_O_3_ (Supplementary Fig. 10) supports after the reaction was mostly {111} plane. The high-resolution transmission electron microscopy (HRTEM) images (Supplementary Fig. 11−14) and the HAADF-STEM images (Fig. 2a−e, Supplementary Figs. 15 and 16) showed the clear presence of very small Ru clusters on the support. In the HAADF-STEM images of Ru/Sm_2_O_3_ (Fig. 2c) and Ru/Al_2_O_3_ (Supplementary Fig. 16c, d), we confirmed that the inter-planar spacing was consistent with the lattice fringe of Ru (100). According to the particle size distribution (Supplementary Figs. 11c, 12c, 13c, 14c, 17) of all catalysts, the size of Ru cluster in all catalysts was mainly 0.5–2 nm, and the average size was 1.2–1.9 nm. The energy dispersive X-ray spectroscopy (EDS) elemental mappings further confirmed the good dispersion of Ru species on the Sm_2_O_3_ (Fig. 2e, Supplementary Fig. 15d) and γ-Al_2_O_3_ (Supplementary Fig. 16e, f). The X-ray diffraction (XRD) patterns (Supplementary Fig. 18) of both the fresh and used catalysts only showed the diffraction peak of the corresponding support, which was consistent with the TEM results because the Ru species were highly dispersed with very low loadings.

The catalytic performances of the Ru-based catalysts (Ru/Sm_2_O_3_, Ru/Y_2_O_3_, Ru/Gd_2_O_3_, Ru/CeO_2_ and Ru/Al_2_O_3_) and corresponding pure oxides materials were evaluated in catalytic ammonia decomposition (Fig. 2f and Supplementary Fig. 19a). This reaction was crucial for online H_2_ production using liquid NH_3_ as the media for H_2_ storage and transportation^19^. First of all, pure metal oxide materials had similar and poor NH_3_ conversion, suggesting a non-catalytic process without the presence of Ru. RE_2_O_3_ supported Ru showed similar activities that were obviously higher than the Ru/Al_2_O_3_, Ru/TiO_2_, Ru/SiO_2_ and Ru/CNTs catalyst (Supplementary Fig. 19b). The NH_3_ conversion of Ru/RE_2_O_3_ catalyst at 450 °C was 60–72%, much higher than that of Ru/Al_2_O_3_ catalyst (only ~13%, GHSV = 30,000 mL·g^−1^·h^−1^). The turnover frequency (TOF) value of Ru/Sm_2_O_3_ (3.2 s^−1^) was 3 times higher than Ru/Al_2_O_3_ (1.1 s^−1^) at 400 °C. The apparent activation energy (*E*_*a*_, Supplementary Fig. 20) of Ru/Sm_2_O_3_ (102.2 kJ·mol^−1^), Ru/Y_2_O_3_ (123.5 kJ·mol^−1^) and Ru/Gd_2_O_3_ (105.1 kJ·mol^−1^) was lower than that of Ru/Al_2_O_3_ (136.2 kJ·mol^−1^), exhibiting superiority in catalytic conversion of NH_3_. Interestingly, even though the *S*_*BET*_ of Ru/RE_2_O_3_ (Supplementary Table 2) was only 1/3–1/2 of Ru/CeO_2_^16^ (*S*_*BET*_ = 106 m^2^·g^−1^), Ru/RE_2_O_3_ catalysts with intrinsic O_v_ had very similar activity to Ru/CeO_2_ with defect-based O_v_. It followed that those intrinsic O_v_ could have similar effects to the defect-based O_v_. In addition, the H_2_ yield with per Ru species of the Ru/RE_2_O_3_ catalysts was 1.5–20 times higher than that of other Ru-based catalysts reported^18,20–32^ (Supplementary Table 3), proving that the Ru-rare earth oxide catalysts achieved the highest noble-metal atom utilization efficiency for ammonia decomposition reaction at present (Fig. 2g). The durability of Ru/Sm_2_O_3_ catalyst was evaluated (Fig. 2h). The NH_3_ conversion of the Ru/Sm_2_O_3_ catalyst decayed by only 4% in 400 h test (GHSV = 36,000 mL·g^−1^·h^−1^). In addition, after six cycles of stability tests, the catalyst still maintained the same NH_3_ conversion at different temperatures (Supplementary Fig. 21), which also verified the excellent stability. We further tested the performance of the Ru/Sm_2_O_3_ catalyst at lower temperatures by the online mass spectrometer (Supplementary Fig. 22). It was found that the catalyst had started catalysing the reaction at a low temperature as 200 °C and had obvious catalytic activity at 250 °C, showing the extraordinary catalytic performance.

**Fig. 3:**
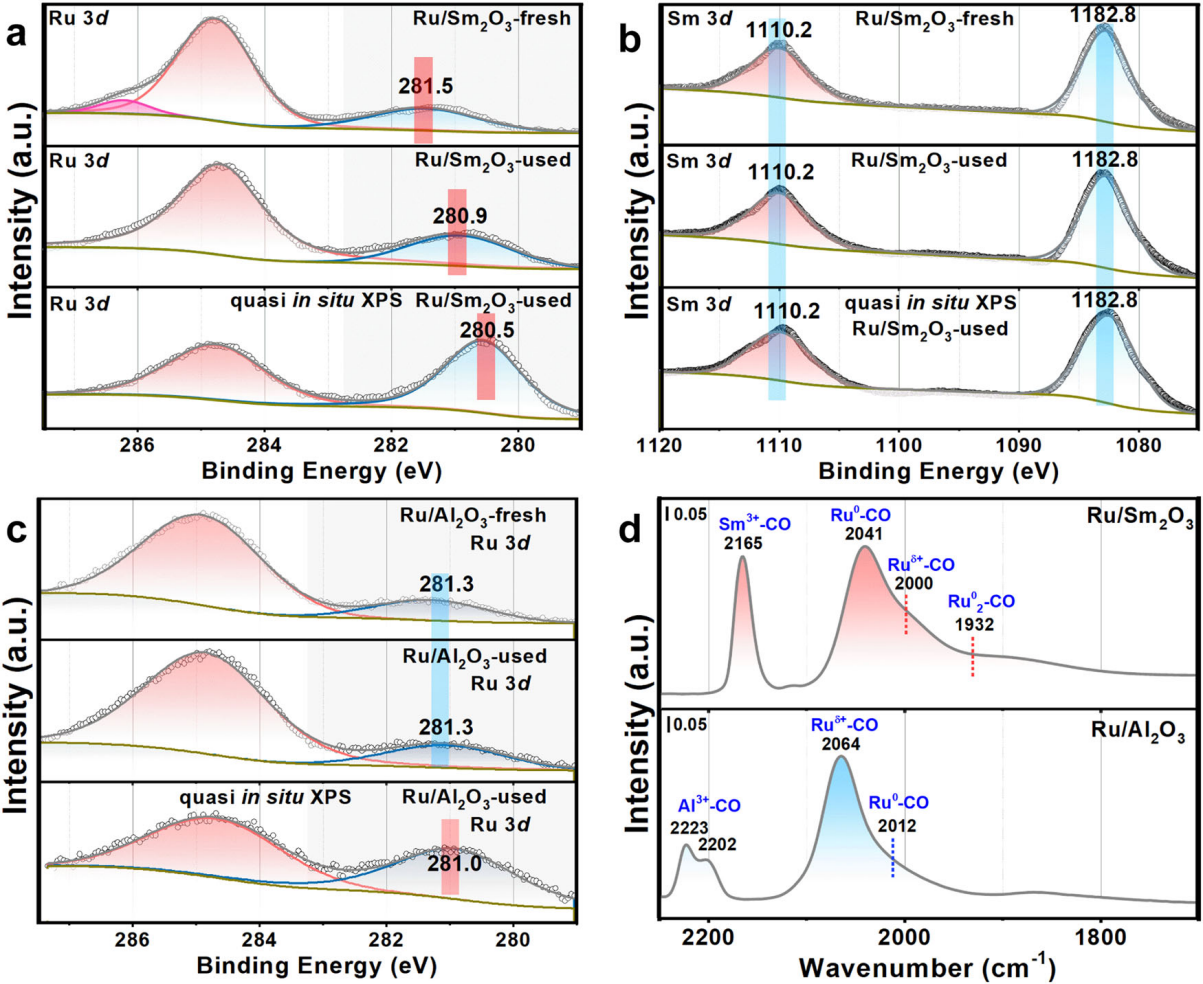
Spectroscopy characterization of catalysts. **a**–**c** XPS results of the fresh and used catalysts: **a** Ru 3*d* of Ru/Sm_2_O_3_, **b** Sm 3*d* of Ru/Sm_2_O_3_, **c** Ru 3*d* of Ru/Al_2_O_3_; **d** the CO adsorption in situ infrared spectroscopy in the transmission mode of used catalysts at 130 K.

The chemical state of the catalyst surface was explored by X-ray photoelectron spectroscopy (XPS, Fig. 3a−c, Supplementary Figs. 23−25 and Supplementary Table 4), Near Edge X-ray absorption fine structure (NEXAFS) (Supplementary Fig. 26) and the in situ infrared (IR) spectroscopy in the transmission mode (Fig. 3d and Supplementary Fig. 27). The initial catalysts contained mainly cationic Ru for all catalysts. After catalysis, most of the cationic Ru in the Ru/Sm_2_O_3_ were reduced to metallic Ru, as shown in the XPS result of the used catalyst and the quasi in situ XPS experiment (Fig. 3a). In comparison, after the same treated process, the Ru on Al_2_O_3_ remained as oxidative state for the used sample (Fig. 3c), only a small amount of Ru was reduced under quasi in situ XPS measurement condition. Such comparison suggested that RE_2_O_3_ surface with intrinsic O_v_ helped the reduction of Ru^δ+^ to Ru^0^ in the presence of H_2_. In the Sm 3*d* (Fig. 3b) and Y 3*d* (Supplementary Fig. 25b) spectra, Sm^3+^ and Y^3+^ were the only species detected, respectively, confirming their non-reducible nature. The surface properties were studied with the Sm M_4,5_ edges and O *K* edge NEXAFS. Both the fresh and used catalysts had exactly the same Sm^3+^ and O features, suggesting a highly durable surface that maintained the intrinsic O_v_ (Supplementary Fig. 26). Comparing to the O *K* edge spectrum of bulk O in Sm_2_O_3_ standard^33^, the surface O in the Ru/Sm_2_O_3_ sample has reduced O 1 s → 5d-π transition comparing to the O 1 s → 5d-σ transition. This showed the slightly different coordination nature between surface and bulk O. To further validate the reduction of Ru, in situ IR experiments during CO adsorption were carried out. The main peak position of Ru/RE_2_O_3_ was concentrated between 2040 and 2050 cm^−1^, which was considered as the CO adsorption on Ru^0^ species^34,35^. While for Ru/Al_2_O_3_ catalysts that were concentrated at 2064 cm^−1^, which was considered as the CO adsorption on Ru^δ+^ species (Fig. 3d). The temperature-programmed reduction by H_2_ (H_2_-TPR, Supplementary Figs. 28 and 29) results showed that Ru on RE_2_O_3_ could be reduced at 123 °C whereas reduction of Ru on Al_2_O_3_ required 271 °C. Combining XPS, NEXAFS, in situ IR and H_2_-TPR, we concluded that Ru over RE_2_O_3_ with intrinsic O_v_ preferred Ru^0^ whereas Ru over Al_2_O_3_ remained at Ru^n+^.

### The catalytic function of RE_2_O_3_ with intrinsic O_v_

The combination of XPS, NEXAFS, IR, DRIFTS and H_2_-TPR study suggested that Ru over RE_2_O_3_ could be easily reduced to Ru^0^ compared with that of Ru/Al_2_O_3_. To validate the catalytic function of RE_2_O_3_ with intrinsic O_v_ in this process and to the NH_3_ decomposition reaction in general, we first used CO_2_-temperature programmed desorption (TPD) to investigate the electrophilic nature of the catalyst (Supplementary Fig. 30). Ru/Sm_2_O_3_ surface contained more medium base sites compared to Ru/Al_2_O_3_, indicating that Ru/Sm_2_O_3_ had more effective surface basicity^29^. This medium basicity was conducive to the electron transfer from Sm_2_O_3_ to Ru species, and further facilitated the dissociative adsorption of N species at Sm^3+^ site. The results of NH_3_-TPD (Supplementary Fig. 31) further proved the NH_3_ desorption was significantly less than that of Ru/Al_2_O_3_, which was consistent with the calculation result of adsorption energy values of NH_3_ molecules on the surface of catalysts (Supplementary Table 1).

**Fig. 4:**
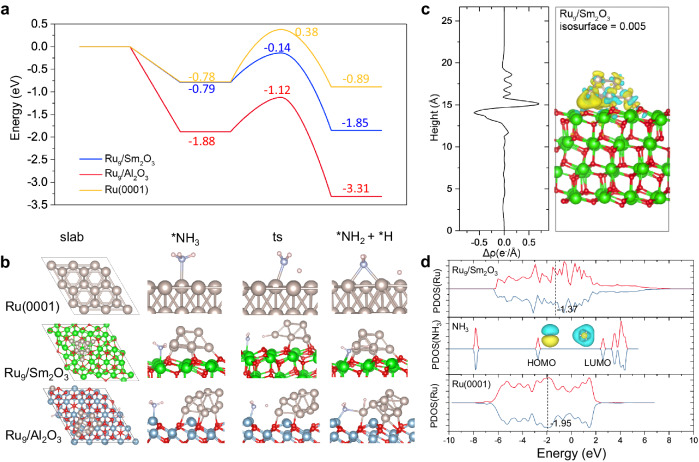
Theoretical results for the reaction mechanism and electronic nature of the active sites. **a** Energy profiles and **b** corresponding structures for NH_3_ adsorption and activation of N–H bond on Ru(0001), Ru_9_/Sm_2_O_3_(111), and Ru_9_/Al_2_O_3_(111), respectively; **c** charge density differences, ∆ρ = ρ(Ru_9_/Sm_2_O_3_) − ρ(Ru_9_) − ρ(Sm_2_O_3_), for Ru_9_ adsorption on Sm_2_O_3_(111) surface (yellow and blue isosurfaces denote where electronic density increase and decrease, respectively) and integration of ∆ρ in planes parallel to the surface and plotted as a function of the z coordination; (d) the projected density of states (PDOS) of the Ru_9_ on Ru_9_/Sm_2_O_3_, isolated NH_3_, and Ru(0001) surface (black dash lines label the *d*-band centre of Ru).

To explore the reaction mechanism, we performed first-principles theoretical calculations (Fig. 4) to further study the N–H dissociation. A Ru (0001) slab was adopted to simulate the large-size Ru nanoparticles while Ru_9_ clusters supported on Sm_2_O_3_(111) slab and γ-Al_2_O_3_(111) were used to simulate the supported cluster catalyst (Ru/Sm_2_O_3_ and Ru/Al_2_O_3_). As shown in Fig. 4a, the N-H bond activation barrier on Ru (0001) surface was 1.16 eV, in good agreement with previous work^36^. Comparatively, the activation barrier of N-H bond on Ru_9_/Al_2_O_3_ and Ru_9_/Sm_2_O_3_ was lowered to 0.76 eV and 0.65 eV, respectively. From Fig. 4b, we could see that on both Ru_9_/Sm_2_O_3_ and Ru_9_/Al_2_O_3_, the N-H dissociation went through a synergistic process that NH_3_ adsorbed on the metal cations in the support surface via the formation N-Sm or N-Al interaction. Meanwhile, H atom in NH_3_ was captured by the Ru cluster, thus resulting in the N-H bond breaking. So, for Ru_9_/Sm_2_O_3_, the intrinsic O_v_ sites were responsible for adsorption of NH_3_, and the interface Ru atoms played the role of activating N-H bonds. After the first N-H bond break, compared to Ru_9_/Sm_2_O_3_ maintained a relatively active state (−1.85 eV), Ru_9_/Al_2_O_3_ was in a very stable state (−3.31 eV), making it difficult for subsequent reactions to occur. As shown in Fig. 4c, the calculated charge density difference showed that electrons transferred from Sm_2_O_3_ slab to Ru_9_ (Supplementary Fig. 32). This result suggested that Sm_2_O_3_ was alkalescence and increased the electron density of Ru to dissociate NH_3_ more favourably. We further calculated the projected density of states (PDOS) of the Ru_9_/Sm_2_O_3_, isolated NH_3_, and Ru (0001) surface (Fig. 4d). To activate the N-H bonds, the *d*-band of Ru and LUMO of NH_3_ must be at similar energy level, which meant Ru metals with higher *d*-band centre will interact with NH_3_ stronger^37^. The *d*-band centres of Ru_9_ on Sm_2_O_3_ and Ru (0001) slab were −1.37 eV and −1.95 eV, respectively, which accounts for the higher activity of Ru/Sm_2_O_3_. The excellent activity of Ru/Sm_2_O_3_ was partially from the sintering-resistant property of Sm_2_O_3_ substrate. The calculated bind energies of Ru_9_ with Al_2_O_3_ and Sm_2_O_3_ were −9.28 eV and −10.24 eV, respectively. Benefiting from the abundant intrinsic surface O_v_ sites in Sm_2_O_3_ surface, Ru clusters were held firmly, thus avoiding the coalescence process and exhibiting solid durability^38,39^.

Based on the present results, the effects of the RE_2_O_3_ with intrinsic surface O_v_ structure for catalysis could be summarized as the following. Firstly, the RE_2_O_3_ with intrinsic surface O_v_ promoted the adsorption and activation of the catalyst on Lewis basic reactant molecules to improve the catalytic performance; secondly, the RE_2_O_3_ with intrinsic surface O_v_ enhanced the interaction between the active metals and the rare earth oxide supports, raising the *d* band of Ru and increase the energy of surface adsorbed NH_2_. To explore the generality of this effect, we prepared Cu/RE_2_O_3_ and Cu/Al_2_O_3_ catalysts and tested the activity of the water-gas shift (WGS) reaction (Supplementary Fig. 33), which was also crucial in industrial hydrogen production. The activity of Cu/RE_2_O_3_ has been found to be significantly higher than that of Cu/Al_2_O_3_, possibly due to the activation of H_2_O and O-H cleavage via intrinsic O_v_.

## Discussion

In summary, we have discovered a new type of O_v_ on the surface of RE_2_O_3_. Those O_v_ stem from the surface symmetric and the atomic arranges and therefore intrinsic of a crystalline, which is completely different from the conventional defect-based O_v_. Such RE_2_O_3_ with intrinsic O_v_ is found to play an important role in catalysis, such as ammonia decomposition and WGS reaction. The RE_2_O_3_ offers significant advantages, including: (1) moderate adsorption of reaction molecules such as NH_3_ and H_2_O; (2) maintaining active species in metallic state, (3) forming unique RE-N(O)-H-Ru configurations for the N(O)-H bond breaking. Such O_v_ -metal synergy is new for those redox inactive metal oxide supports and will bring RE_2_O_3_ on the screening system for heterogenous catalysis.

## Methods

### Synthesis of catalysts

The typical synthetic method of Ru colloidal solution has been reported previously^16^. Dissolving 0.15 g RuCl_3_ (Sinopharm) in 50 mL ethylene glycol (C_2_H_6_O_2_; Sinopharm), and then added 0.16 g NaOH (Sinopharm) to the mixture with constant stirring for 30 min. Next, the solution was refluxed at 160 °C for 3 h. After cooling to room temperature, we obtained the Ru colloidal solution with dark brown.

Sm_2_O_3_ nanorod (Sm_2_O_3_) and CeO_2_ nanorod (CeO_2_) follow the same hydrothermal method. Dissolving NaOH (14.40 g; Sinopharm) in 40 mL deionized water, and then the solution of 3 mmol nitrate (Sm(NO_3_)_3_·6H_2_O (aladdin) and Ce(NO_3_)_3_·6H_2_O (kermel)) was added into the previous mixture and kept stirring for 30 min. Then we transferred the solution to the teflon bottle for hydrothermal reaction at 100 °C for 24 h. The precipitate produced was washed and dried overnight at 60 °C to obtain Sm(OH)_3_ and CeO_2_. The Sm(OH)_3_ was calcined in air at 450 °C for 4 h to obtain Sm_2_O_3_.

Y_2_O_3_ nanosheet was synthesized by hydrothermal method. Dissolving 1.149 g Y(NO_3_)_3_·6H_2_O (aladdin) in 60 mL deionized water. And then using 10% NaOH solution adjusted the pH to 12. Then we transferred the solution to the teflon bottle for hydrothermal reaction at 120 °C for 12 h. The precipitate produced was washed and dried overnight at 60 °C. Finally, it was calcined in the air at 500 °C for 6 h.

Gd_2_O_3_ nanorod was synthesized by hydrothermal method. Dissolving 0.02 mol Gd(NO_3_)_3_·6H_2_O (aladdin) in 50 mL deionized water. And then using 2.5 mol·L^−1^ NaOH solution adjusted the pH to 12.8. Then we transferred the solution to the teflon bottle for hydrothermal reaction at 180 °C for 24 h. The precipitate produced was washed and dried overnight at 80 °C. Finally, it was calcined in the air at 450 °C for 2 h.

The Ru-based catalysts were synthesized according to the previous methods^16^. The typical steps of colloidal deposition method were shown as followed. 1 g supports (Sm_2_O_3_, CeO_2_, Y_2_O_3_, Gd_2_O_3_ and γ-Al_2_O_3_ (commercial, Macklin)) was dissolved and dispersed in 25 mL deionized water, and then added a certain amount of Ru colloid to this mixture and kept stirring for 48 h. Next, ageing for 12 h and the precipitates were collected and washed by centrifugation. The obtained products were dried for 48 h at 60 °C. Finally, the catalysts were reduced in NH_3_ atmosphere at 550 °C before catalytic test.

### Characterization of catalysts

The inductively coupled plasma mass spectrometer (ICP-MS, PerkinElmer, NexION 350X) analysis was used to detect the actual content of Ru. The N_2_ adsorption−desorption measurements were on a Builder SSA-4200 analyzer at −196 °C. All the samples were pretreated at 200 °C for 400 min under vacuum. The BET (according to the Brunauer, Emmett and Teeler method) specific surface area (*S*_*BET*_) can be calculated from that. The X-ray diffraction (XRD) was carried out on a PANalytical X’pert3 powder diffractometer (40 kV, 40 mA) using Cu Kα radiation (λ = 0.15406 nm). The diffraction angles (2*θ*) ranged from 10° to 90°. The thermogravimetric analysis (TGA) was carried out on a simultaneous thermal analyzer (METTLER, TGA/DSC3 + ) in N_2_. The transmission electron microscopy (TEM) images were conducted on a JEOL JEM-2100F microscope operating at 100 kV. The high-resolution TEM (HRTEM) was carried out under 200 kV on a FEI Talos F200s microscope. The high-angle annular dark-field scanning transmission electron microscopy (HAADF-STEM) images were obtained on a JEOL ARM200F microscope equipped with a probe-forming spherical-aberration corrector and Gatan image filter (Quantum 965). The hydrogen temperature-programmed reduction (H_2_-TPR) measurements were carried on a Builder PCSA-1000 instrument. After pretreated at 500 °C in air, 50 mg catalysts were reduced by 5%H_2_/Ar (30 mL·min^−1^) from room temperature to 700 °C. The H_2_ consumption was shown by the thermal conductivity detector (TCD). The X-ray photoelectron spectroscopy (XPS) measurements were carried out on an Thermo scientific ESCALAB Xi^+^ XPS spectrometer with Al Kα radiations, and with the C 1 *s* peak at 284.8 eV as an internal standard for all the spectra. The in situ infrared spectroscopy in the transmission mode measurements were conducted in a UHV apparatus combining a FTIR spectrometer (Bruker Vertex 70 v) with a multi-chamber UHV system. The sample was pretreated with H_2_ in a vacuum at 873 K for 30 min, and then exposed to CO (10^−2^ mbar) at 130 K to collect the spectrogram.

Near Edge X-ray absorption fine structure (NEXAFS) experiments were carried out at the VerSoX beamline (B07-C) of Diamond Light Source (DLS, UK). The beamline has a maximum resolving power hν/Δ(hν) > 5000 with a photon flux > 10^10^ photons s^−1^ from 170 eV to 2000 eV and can be operated (delivering lower flux) up to 2800 eV. The accuracy of the sample and analyser position is typically less than 10 µm. The gas pressure and composition are controlled via a butterfly valve and mass flow controllers. The endstation consists of a fixed interface flange which holds the entrance cone of the ambient-pressure electron energy analyser (SPECS Phoibos NAP-150). The samples (around 1 mg) were dispersed in water (around 1 mL) and dropped (around 2 droplets) on Au-coated Si (~1 cm × 1 cm), followed by heating at 70 °C to remove the solvent. NEXAFS spectra at Sm M_4_/M_5_ edge and O K-edge were measured in both total electron yield (TEY) mode and Auger electron yield (AEY) mode at room temperature. The measurements were performed under UHV condition.

The temperature programmed desorption (TPD) measurements of the catalysts were performed at the online mass spectrometer (TILON LC-D200M). For the typical NH_3_-TPD experiments, 300 mg catalysts (20–40 mesh) were pretreated at 550 °C for 1 h in NH_3_ atmosphere (20 mL·min^−1^). And then it was cooled to room temperature, and held for 1 h in the NH_3_ atmosphere. Next, switched to Ar and purged until the baseline was stable, collected the signal from room temperature to 800 °C. For the typical CO_2_-TPD experiments, 300 mg catalysts (20–40 mesh) were pretreated at 550 °C for 1 h in 5%H_2_/Ar (30 mL·min^−1^). And then it was cooled to room temperature, and held for 1 h in the CO_2_ atmosphere. Next, switched to Ar and purged until the baseline was stable, collected the signal from room temperature to 800 °C. For the reaction at lower temperatures, 50 mg catalysts (20–40 mesh) were pretreated at 550 °C for 1 h in NH_3_ atmosphere (20 mL·min^−1^). After cooling down, we collected the signal from 100 °C to 300 °C with a step of 50 °C in continuous pure NH_3_ flow.

### Theoretical methods and computational details

All static calculations were carried out using spin-polarized density functional theory (DFT) with generalized gradient approximation (GGA) of Perdew–Burke–Ernzerhof (PBE) and PAW pseudopotentials as implemented in VASP 5.4.4 code^40,41^. DFT + U method with U = 4 eV was used to describe the localized rare earth 4 *f* states^42^. The valence orbitals were described by plane-wave basis sets with a cutoff energy of 400 eV. Considering the large size of p(4×4)-(111) slabs used in this work, the single gamma-point grid sampling was used for Brillouin Zone integration for geometry optimization, and 3 × 3 × 1 k-mesh was used for density of states calculations. Atomic positions were optimized by using the conjugate gradient algorithm until the forces were less than 0.03 eV/Å. Transition states were searched by climbing image nudged-elastic-band (CI-NEB) method with convergence criterion of 0.05 eV/Å^43,44^. The criterion for electronic self-consistent field convergence was set to 10^−6^ eV.

### Catalytic tests

The catalytic performance of the catalysts was tested in a self-constructed fixed-bed flow reactor. The temperature controller (UDIAN, XIAMEN YUDIAN AUTOMATION TECHNOLOGY CO., LTD.) was used in the reactor temperature control system. During the test, 50 mg catalysts (20–40 mesh) mixed with 500 mg quartz sand (20–40 mesh) and then packed into the reactor with an inner diameter of 6 mm. Before the test, the catalysts were activated at 550 °C in pure NH_3_ atmosphere. Then the activity test was performed from 350 to 550 °C with a step of 50 °C for the reactor temperature (GHSV = 30,000 mL·g^−1^·h^−1^). The outlet gas was analyzed by an online gas chromato-graph (Ouhua GC 9160), and then the real-time N_2_ and NH_3_ contents were obtained. The NH_3_ conversion was calculated through Eq. (1).

1 $${{X}}_{{{{{{{\rm{NH}}}}}}}_{3}}=\frac{2\times {n}_{{{{{{{\rm{N}}}}}}}_{2}}^{{{{{{\rm{out}}}}}}}}{2\times {n}_{{{{{{{\rm{N}}}}}}}_{2}}^{{{{{{\rm{out}}}}}}}+{n}_{{{{{{{\rm{NH}}}}}}}_{3}}^{{{{{{\rm{out}}}}}}}}\times 100\%$$

The stability test of the catalysts was conducted at 450 °C (GHSV = 36,000 mL·g^−1^·h^−1^) for 400 h. The apparent activation energy for the reaction was determined with an equal conversion of 12.5% by tuning the flow rate and temperature.

### Supplementary information

Supplementary Information

Peer Review File

### Source data

Source Data

## Data Availability

The main data supporting the findings of this study are available within the article and its Supplementary Information. Additional data are available from the corresponding authors upon request. Source data are provided with this paper.
