# Supplementary material for: Catalytic properties of trivalent rare-earth oxides with intrinsic surface oxygen vacancy
Source: Nat Commun. 2024 Jul 9;15:5751. doi: 10.1038/s41467-024-49981-9 (PMC11233603; doi:10.1038/s41467-024-49981-9)
Supplement: Supplementary file 1 — Supplementary Information [file 41467_2024_49981_MOESM1_ESM.pdf]

Supplementary Information for

Catalytic properties of trivalent rare-earth oxides with intrinsic surface oxygen vacancy

Kai Xu[1‡], Jin-Cheng Liu[2,3‡], Wei-Wei Wang[1], Lu-Lu Zhou[1], Chao Ma[4], Xuze Guan[5], Feng Ryan Wang[5]*, Jun Li[2,6]*, Chun-Jiang Jia[1]*, Chun-Hua Yan[7]

[1]Key Laboratory for Colloid and Interface Chemistry, Key Laboratory of Special Aggregated Materials, School of Chemistry and Chemical Engineering, Shandong University, Jinan 250100, China.
[2]Department of Chemistry and Engineering Research Center of Advanced Rare-Earth Materials of Ministry of Education, Tsinghua University, Beijing 100084, China.
[3]Center for Rare Earth and Inorganic Functional Materials, School of Materials Science and Engineering & National Institute for Advanced Materials, Nankai University, Tianjin 300350, China.
[4]College of Materials Science and Engineering, Hunan University, Changsha, 410082, China.
[5]Department of Chemical Engineering, University College London, Roberts Building, Torrington Place, London WC1E 7JE, UK.
[6]Fundamental Science Center of Rare Earths, Ganjiang Innovation Academy, Chinese Academy of Sciences, Ganzhou 341000, China.
[7]Beijing National Laboratory for Molecular Sciences, State Key Lab of Rare Earth Materials Chemistry and Applications, PKU-HKU Joint Lab in Rare Earth Materials and Bioinorganic Chemistry, Peking University, Beijing 100871, China.

[‡]These authors contributed equally: Kai Xu, Jin-Cheng Liu.

*Corresponding author. Email: ryan.wang@ucl.ac.uk, junli@mail.tsinghua.edu.cn, jiacj@sdu.edu.cn.

Table of Contents

Supplementary Methods

Supplementary Figures and Supplementary Notes

Supplementary Tables

Supplementary References

Supplementary Methods:

Preparation of Cu/RE_2O_3 and Cu/Al_2O_3 catalysts. The catalysts were prepared by deposition-precipitation (DP) method. The support (0.05 g) dispersed in high purity water (30 mL) by stirring and ultrasound. Meanwhile, a specific mass of $Cu(NO_3)_2$ was dissolved in high purity water to prepare an aqueous Cu solution. Where the loading of Cu was 5 wt% (5 wt% = [Cu/support] wt × 100%). Then, the Cu solution was added drop by drop to the above support/H_2O suspension dropwise. The pH of the solution was maintained at 9 by using Na_2CO_3 solution (0.50 mol·L^{-1}) during the process. Next, the mixture was stirred for 0.5 h and aged for 1 h. The precipitate was obtained by filtration and washed. The resulting material was dried in air at 75 °C overnight and then calcined in still air at 400 °C for 4 h (heating rate: 2 °C·min^{-1}). Finally, we obtained the Cu/Y_2O_3, Cu/Gd_2O_3, Cu/CeO_2 and Cu/Al_2O_3 catalysts.

Catalytic performance test for water-gas shift (WGS) reaction. The activity of catalysts was tested in a self-constructed fixed-bed flow reactor. The temperature controller (UDIAN, XIAMEN YUDIAN AUTOMATION TECHNOLOGY CO., LTD.) was used in the reactor temperature control system. Both the actual temperature of the furnace temperature and the location of the catalyst were detected. The actual reaction gas content was 2%CO, 10%H_2O, and the equilibrium gas was N_2. During the test, 25 mg catalysts (20−40 mesh) were filled with gas hourly space velocity (GHSV) was 168,000 mL·g^{-1}·h^{-1}. The catalysts were pretreated in a 5% H_2/N_2 at 300 °C. After a cooling system, the dry gas entered the gas analyzer (Gasboard-3100, Wuhan Sifang Corp), and then the real-time CO and CO_2 contents were obtained. The CO conversion was calculated through eq. (1).

$$X_{\mathrm{CO}} = \frac{n_{\mathrm{CO}}^{\mathrm{in}} - n_{\mathrm{CO}}^{\mathrm{out}}}{n_{\mathrm{CO}}^{\mathrm{in}}} \times 100\% \qquad (1)$$

Supplementary Figures and Supplementary Notes:

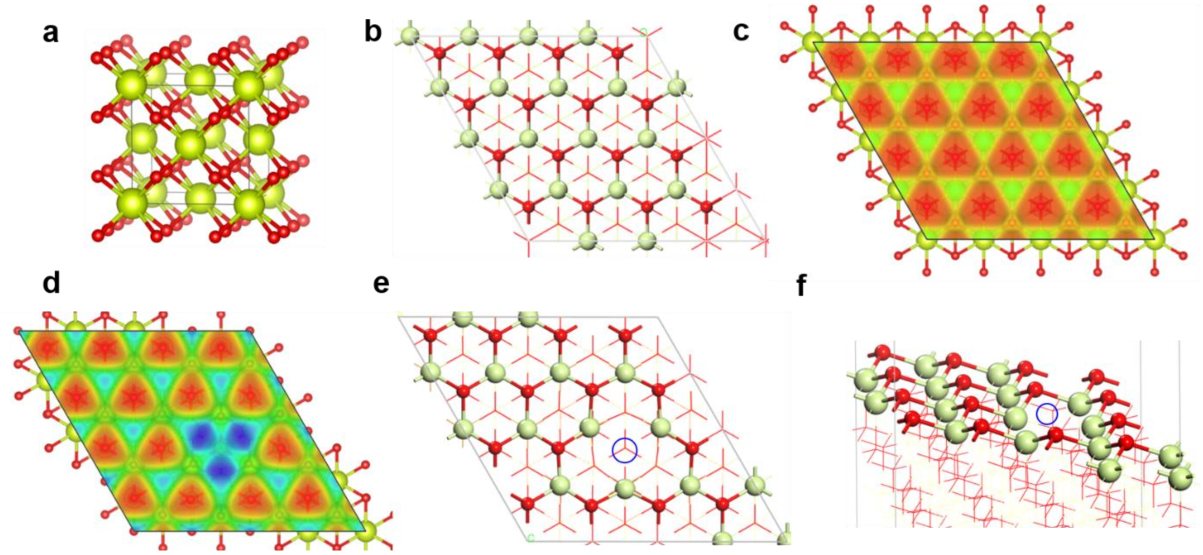

Supplementary Fig. 1 | Theoretical model of CeO_2. (a) The crystal structure of CeO_2; (b) the structure of the perfect CeO_2 (111) surface; (c) the electrostatic potential of the perfect CeO_2 (111) surface; (d) electron density isosurface mapped with electrostatic potential surface of the reduced CeO_2 with surface vacancy (CeO_2-v (111)); (e) top view and (f) side view of optimized surface structure of the reduced CeO_2 with surface vacancy (CeO_2-v (111)). Blue circles indicated oxygen vacancy, blue and orange areas on electrostatic potential surface indicate electrophilic and nucleophilic sites, respectively.

Supplementary Note 1: For CeO_2, which was fluorite structured and crystallizes in $Fm\bar{3}m$ space group, no Ce site was exposed on the perfect O-terminated (111) surface (Supplementary Fig. 2a−c). Meanwhile, because of the reduction of Ce (IV) to Ce (III), the surface oxygen in CeO_2 was released, leading to the exposure of Ce site and formation of oxygen vacancy in (111) (Supplementary Fig. 2d−f) [1,2]. The oxygen vacancies in CeO_2 (111) were electrophilic (Supplementary Fig. 2d), which were ideal sites for adsorption of polarized molecules, such as H_2O and NH_3[3].

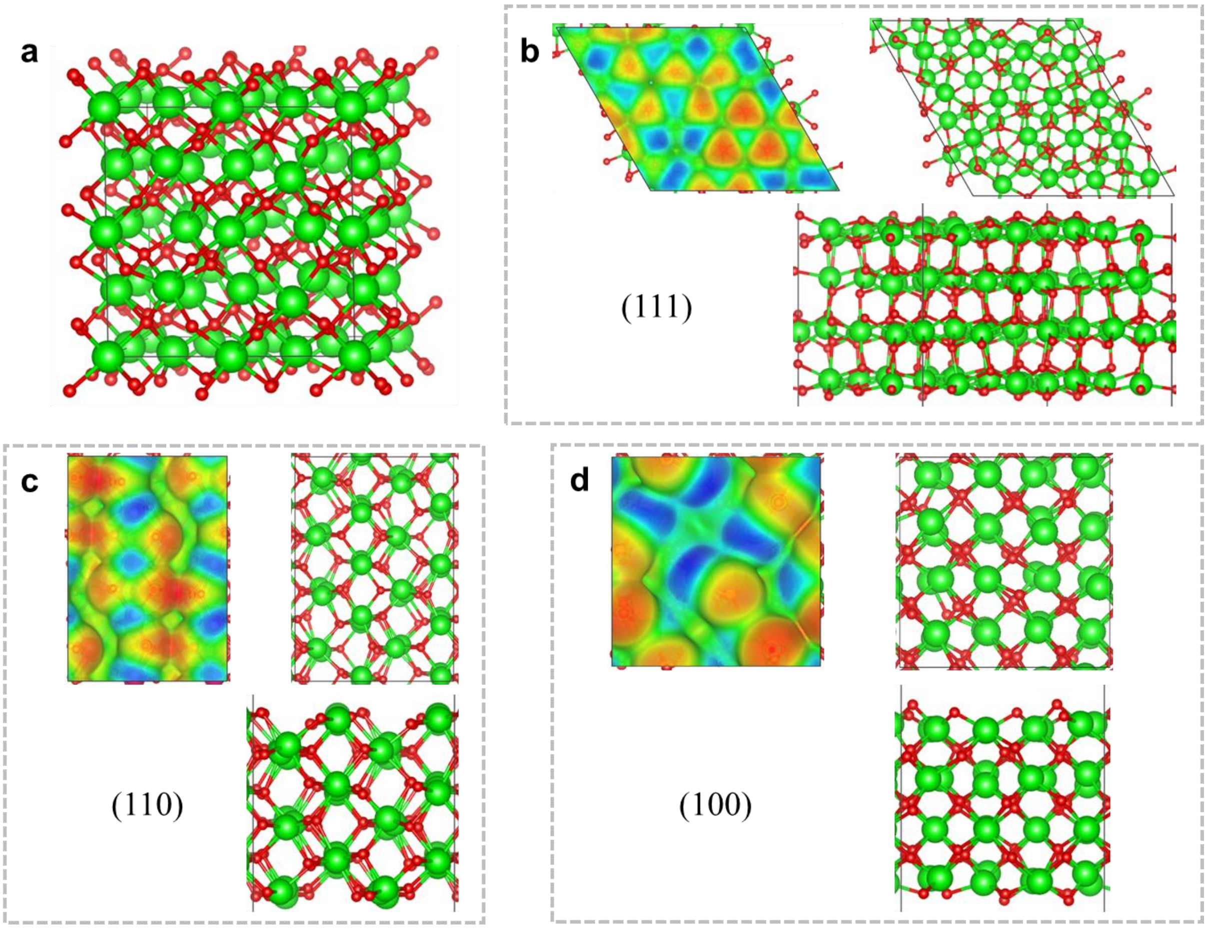

Supplementary Fig. 2 | Theoretical model of Sm_2O_3. (a) The crystal structure of Sm_2O_3; (b) the structure and the electrostatic potential of Sm_2O_3 (111) surface; (c) the structure and the electrostatic potential of Sm_2O_3 (110) surface; (d) the structure and the electrostatic potential of Sm_2O_3 (100) surface. Blue and orange areas on electrostatic potential surface indicate electrophilic and nucleophilic sites, respectively.

Supplementary Note 2: Structural and electrostatic-potential-colored charge density isosurfaces at 0.003 |e|/bohr^3 for Sm_2O_3(110), Sm_2O_3(100) surfaces, where the orange region was dominated by electrons and the blue region was dominated by nuclei. There were many electrophilic sites on the surface, which were similar to the properties of oxygen vacancy (O_v) of CeO_2 surface[3].

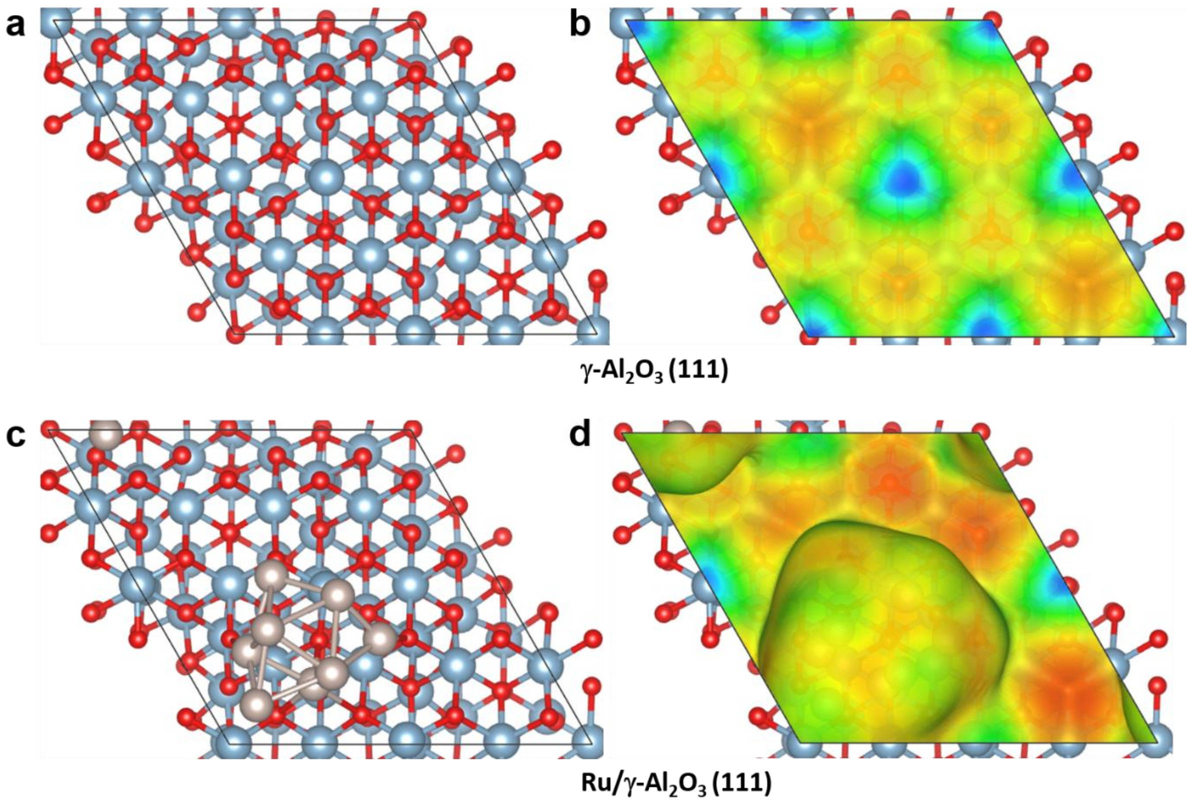

Supplementary Fig. 3 | Theoretical model of γ-Al_2O_3. (a) The structure of the γ-Al_2O_3 (111) surface; (b) the electrostatic potential of the γ-Al_2O_3 (111) surface; (c) the structure of the Ru_9/γ-Al_2O_3 (111) surface; (d) the electrostatic potential of the Ru_9/γ-Al_2O_3 (111) surface. Blue and orange areas on electrostatic potential surface indicate electrophilic and nucleophilic sites, respectively.

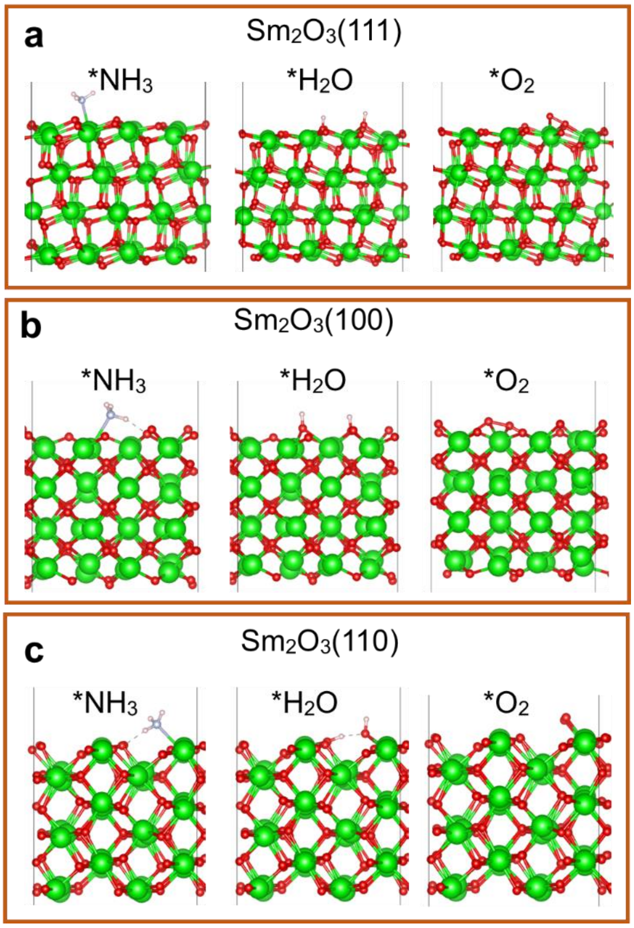

Supplementary Fig. 4 | Model of molecules (NH_3, H_2O and O_2) adsorption on the Sm_2O_3 surface. (a) Sm_2O_3(111) surface; (b) Sm_2O_3(100) surface; (c) Sm_2O_3(110) surface.

Supplementary Note 3: In order to comprehensively disclose the effects of different planes on catalytic activity, the adsorption energy of three typical molecules (NH_3, H_2O and O_2) on the various surfaces of Sm_2O_3 are investigated. The moderate adsorption of NH_3 on the Sm_2O_3(111) surface (−0.44 eV) was stronger than the Sm_2O_3(110) surface (−0.36 eV), but weaker than that on the Sm_2O_3(100) surface (−0.98 eV). These results suggest the Sm_2O_3(111) exhibits moderate adsorption strength among the investigated surfaces, which also demonstrates the rationalization of the theoretical model that we selected. The calculation results of adsorption energy about H_2O and O_2 also shows the same trend. In addition, even NH_3 adsorbed on Sm_2O_3(100) surface (−0.98 eV) is still favourable to the activation of NH_3 molecule than that on γ-Al_2O_3(111) surface, because the latter exhibits an excessively strong adsorption of NH_3 (−1.74 eV). The above result suggests the superiority about activation of electron rich molecules on the surfaces of RE_2O_3.

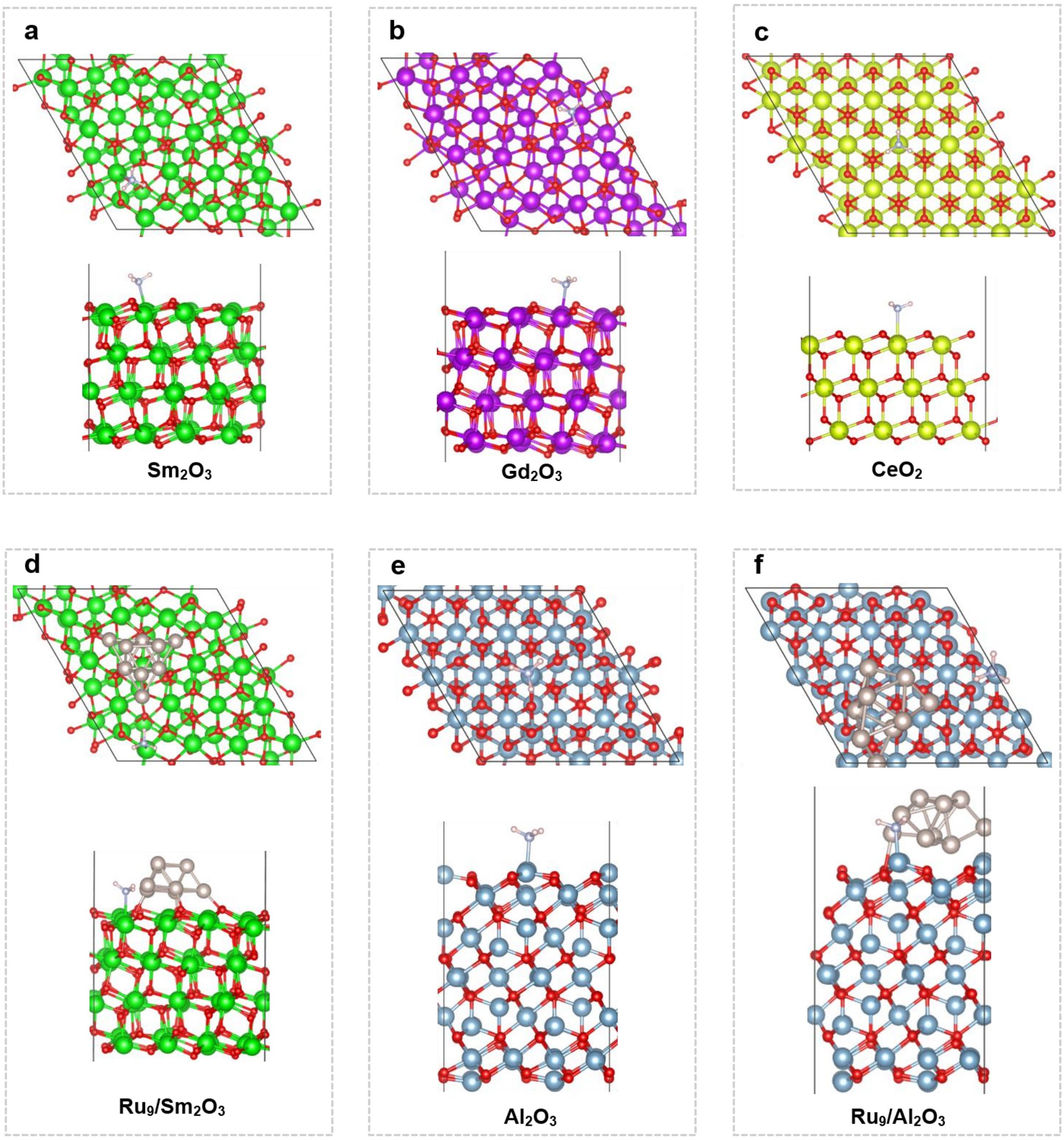

Supplementary Fig. 5 | Model of NH_3 adsorption on the metal oxides surface. (a) Sm_2O_3 (111) surface; (b) Gd_2O_3 (111) surface; (c) CeO_2 (111) surface; (d) Ru_9/Sm_2O_3 surface; (e) Al_2O_3 (111) surface; (f) Ru_9/Al_2O_3 (111) surface.

Supplementary Note 4: The adsorption energy for NH_3 on these oxides was seen in Supplementary Table 1.

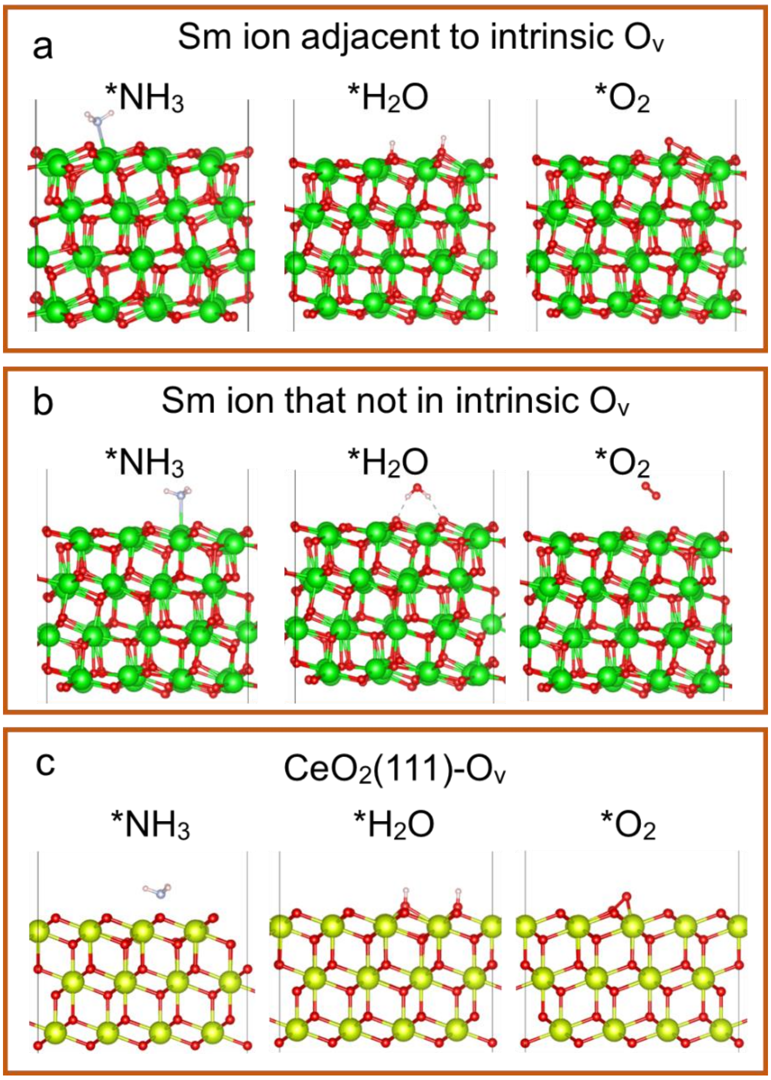

Supplementary Fig. 6 | Model of molecules (NH_3, H_2O and O_2) adsorption on the catalysts surface. (a) Sm ion adjacent to intrinsic O_v; (b) Sm ion that not in intrinsic O_v; (c) CeO_2 with surface vacancy (CeO_2-v (111)).

Supplementary Note 5: The influence of particular spatial structures on molecular adsorption should include geometric effect as well as electronic effect. The activation of molecules is generally achieved by electron transfer between the adsorbed molecules on the surface and the active sites, which further causes structural buckling and a longer bond length. The nucleophilic N atoms in the NH_3 are likely to be adsorbed and activated efficiently at the electrophilic intrinsic oxygen vacancy. In addition, the steric hindrance is presented during the adsorption of the NH_3 (triangular pyramidal molecule). Intrinsic oxygen vacancy might provide favorable space for their adsorption and activation, meeting the requirements of their local coordination environment.

The coordination number of Sm ions adjacent to intrinsic oxygen vacancies on the $Sm_2O_3(111)$ surface is reduced to 5 or 6, as opposed to a full coordination number of 7 in bulk Sm_2O_3. This structural aspect has significant implications for catalysis. Owing to the distinction of Sm ions in spatial position, the Sm ions adjacent to intrinsic O_v and that not in intrinsic O_v might exhibit different coordination environments and charge densities. Such differences will cause the distinction in the adsorption for reactant molecules. Our investigation reveals that these intrinsic oxygen vacancies moderate the adsorption energies of essential reactants (NH_3, H_2O, O_2) differently from both traditional oxygen vacancies in $CeO_2(111)$ and intact Sm_2O_3. The adsorption is not as strong as on $CeO_2(111)$ with vacancies, which can be too robust, leading to difficulties in product desorption. Conversely, it is stronger than on non-vacancy Sm sites of $Sm_2O_3(111)$ surface, where adsorption might be too weak for effective catalysis.

This balanced adsorption strength afforded by the intrinsic oxygen vacancies in Sm_2O_3 is what we refer to as having the potentials for catalysis. It facilitates a catalytic process where product desorption is not hindered by overly strong adsorption, thus potentially enhancing catalytic turnover. This moderation in adsorption strength is pivotal for reactions such as ammonia decomposition, which require a delicate balance between adsorption and desorption to achieve optimal efficiency.

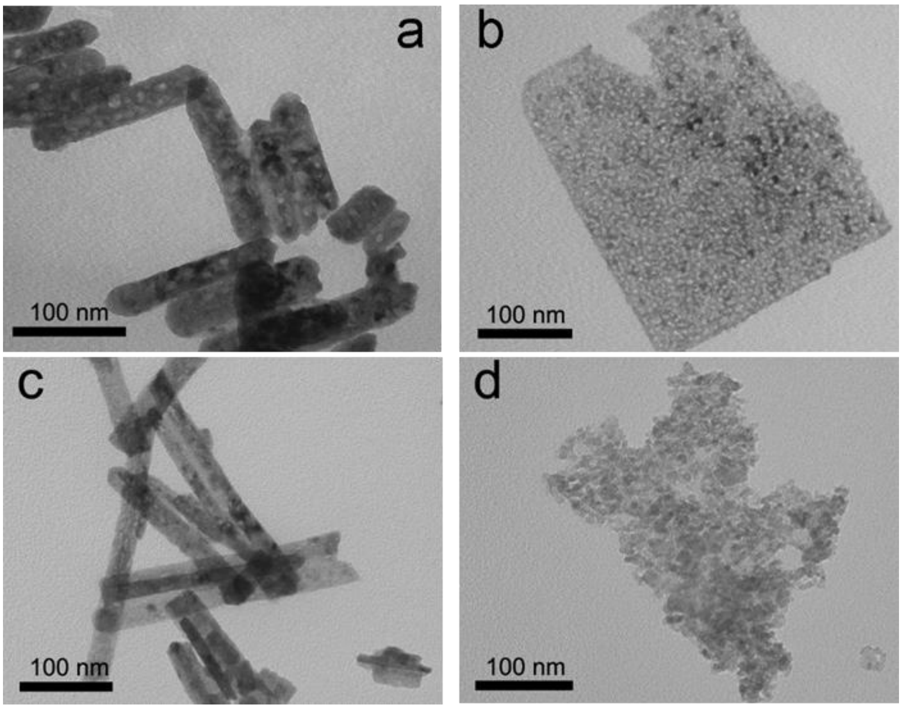

Supplementary Fig. 7 | TEM images of the fresh catalyst. (a) Ru/Sm_2O_3; (b) Ru/Y_2O_3; (c) Ru/Gd_2O_3; (d) Ru/Al_2O_3.

Supplementary Note 6: Supplementary Fig. 7 showed the TEM images of the fresh catalyst. It could be found that there was no obvious Ru species observed. Ru/Sm_2O_3 and Ru/Gd_2O_3 were nanorods, Ru/Y_2O_3 was nanosheet and Ru/Al_2O_3 was nanoparticle.

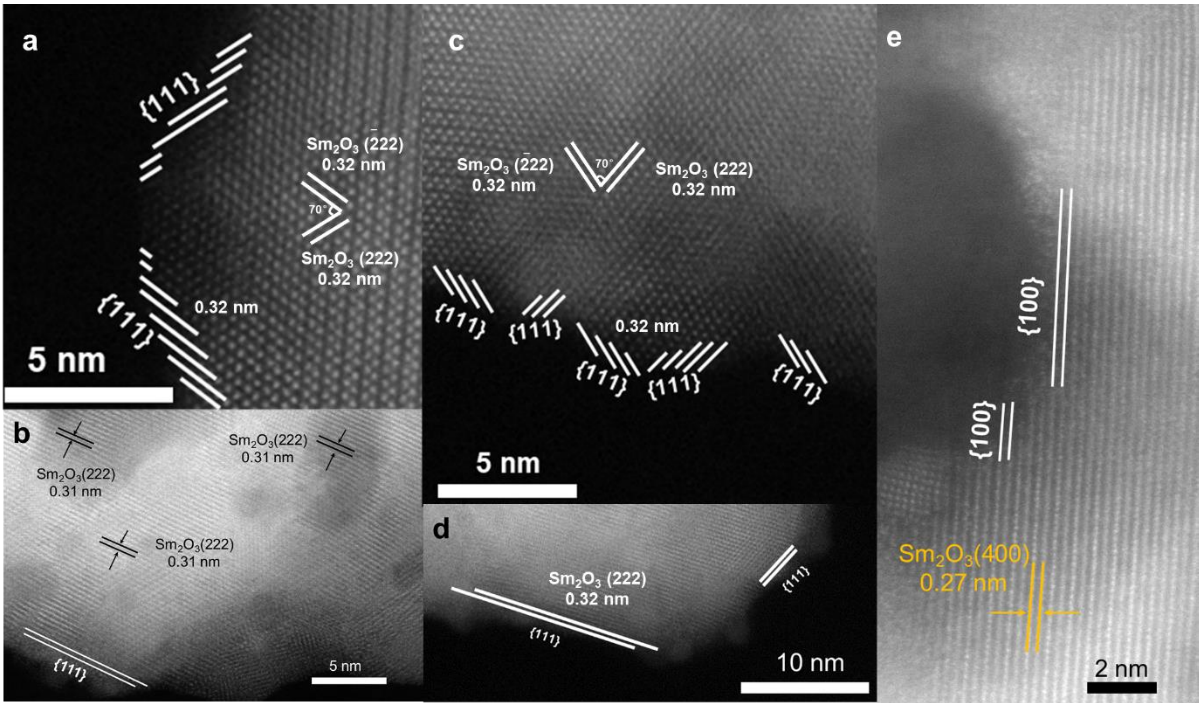

Supplementary Fig. 8 | The aberration-corrected HAADF-STEM images of the used Ru/Sm_2O_3 (a−e).

Supplementary Note 7: A large amount of Sm_2O_3{111} ((222) planes) could be concluded through the analysis of the lattice fringes. Only a few Sm_2O_3{100} ((400) planes) could be observed in the location of the surface, and no corresponding fringes of Sm_2O_3{110} could be found.

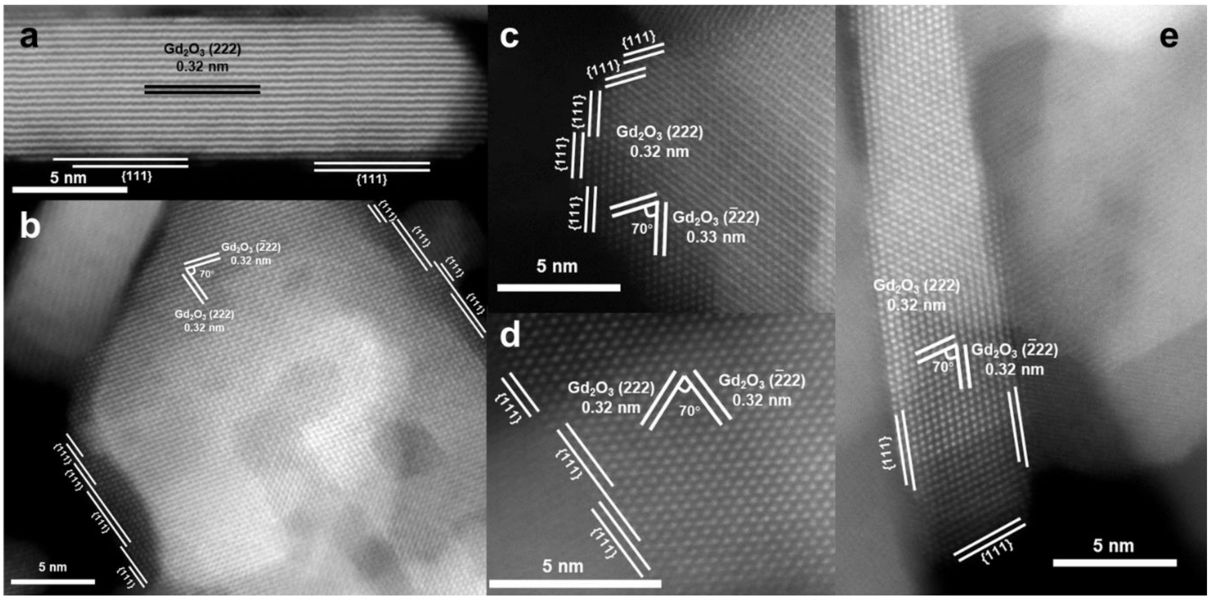

Supplementary Fig. 9 | The aberration-corrected HAADF-STEM images of the used Ru/Gd_2O_3 (a−e).

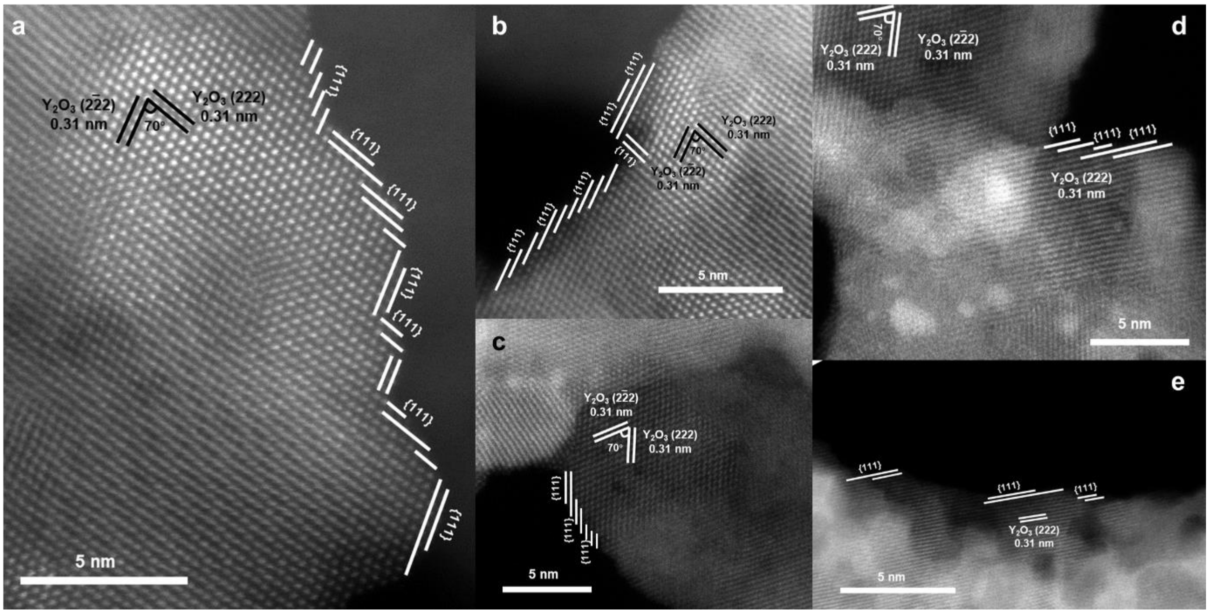

Supplementary Fig. 10 | The aberration-corrected HAADF-STEM images of the used Ru/Y_2O_3 (a−e).

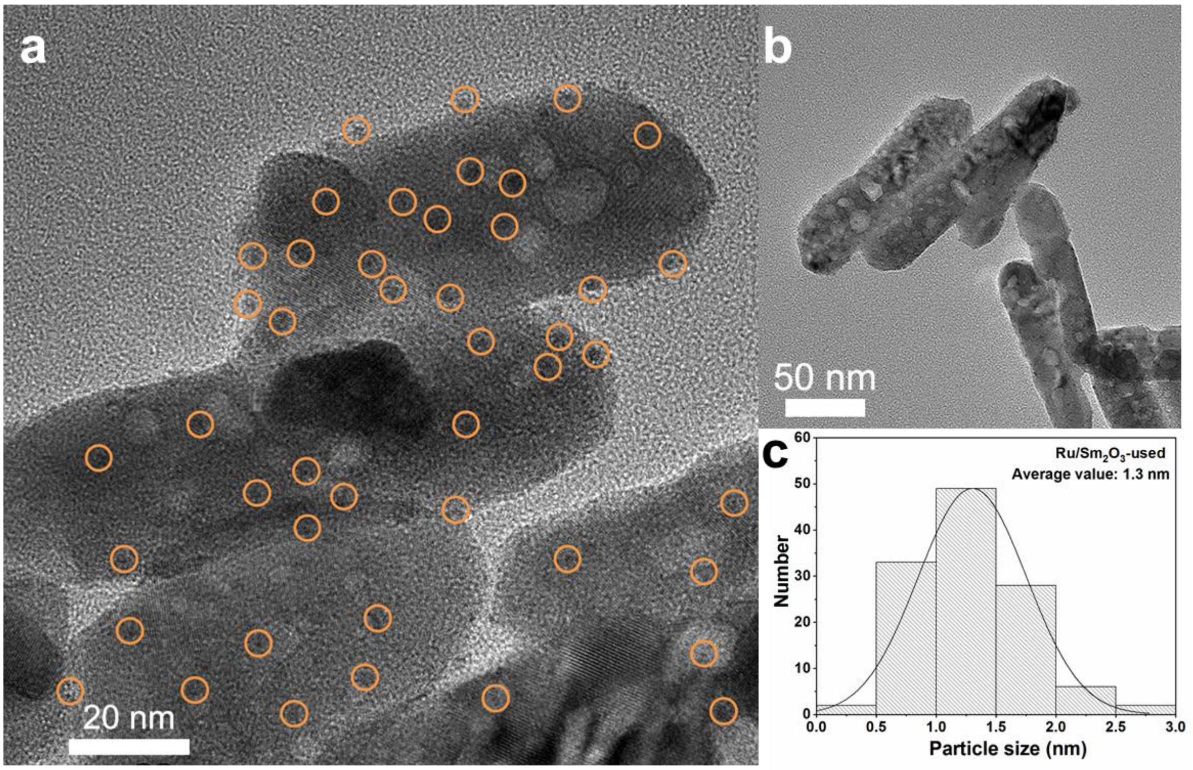

Supplementary Fig. 11 | HRTEM images of the used Ru/Sm_2O_3. (a, b) HRTEM images of the Ru/Sm_2O_3-used catalyst; (c) the particle size distribution of Ru species for Ru/Sm_2O_3-used catalyst.

Supplementary Note 8: The average particle size of Ru clusters on Ru/Sm_2O_3-used catalyst was 1.3 nm.

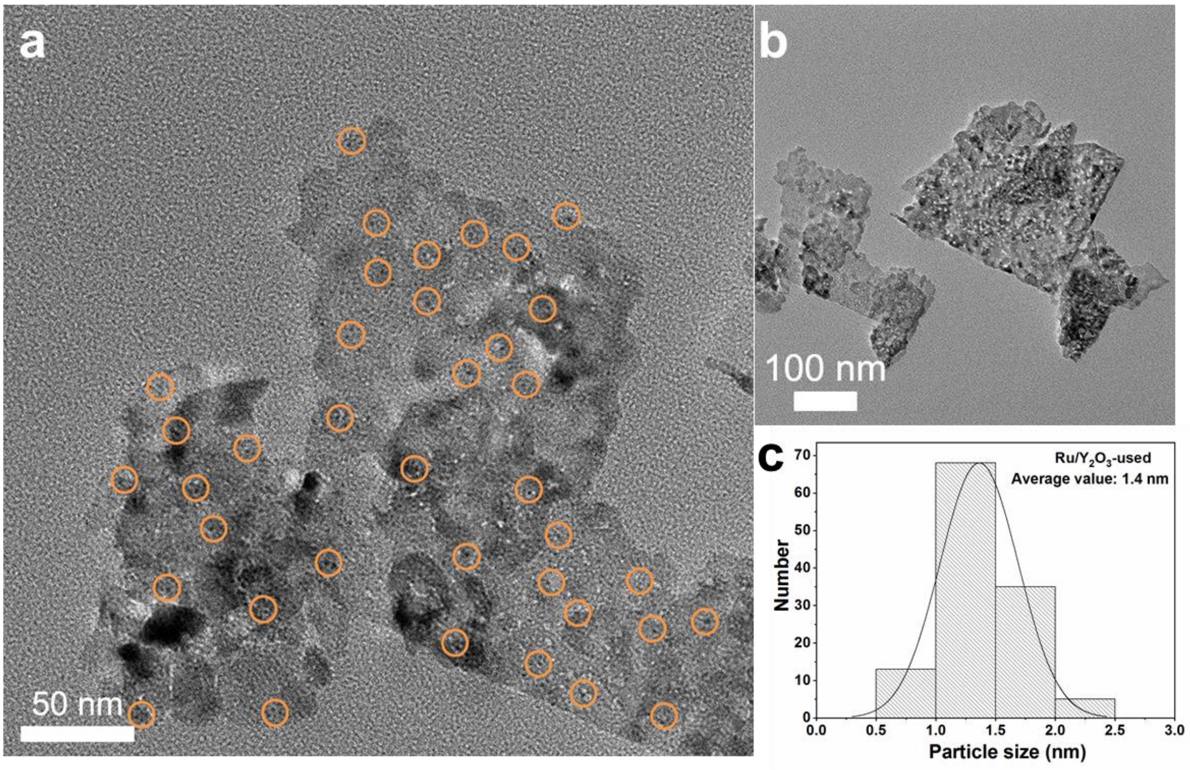

Supplementary Fig. 12 | HRTEM images of the used Ru/Y_2O_3. (a, b) HRTEM images of the Ru/Y_2O_3-used catalyst; (c) the particle size distribution of Ru species for Ru/Y_2O_3-used catalyst.

Supplementary Note 9: The average particle size of Ru clusters on Ru/Y_2O_3-used catalyst was 1.4 nm.

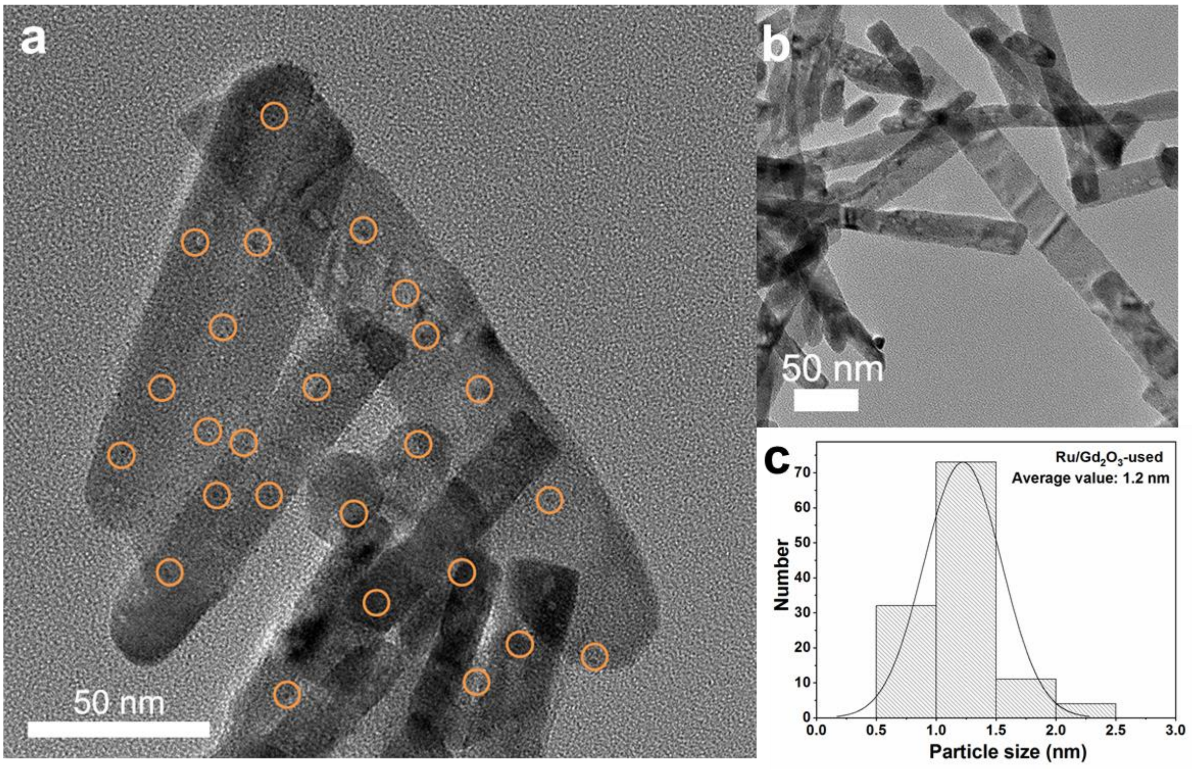

Supplementary Fig. 13 | HRTEM images of the used Ru/Gd_2O_3. (a, b) HRTEM images of the Ru/Gd_2O_3-used catalyst; (c) the particle size distribution of Ru species for Ru/Gd_2O_3-used catalyst.

Supplementary Note 10: The average particle size of Ru clusters on Ru/Gd_2O_3-used catalyst was 1.2 nm.

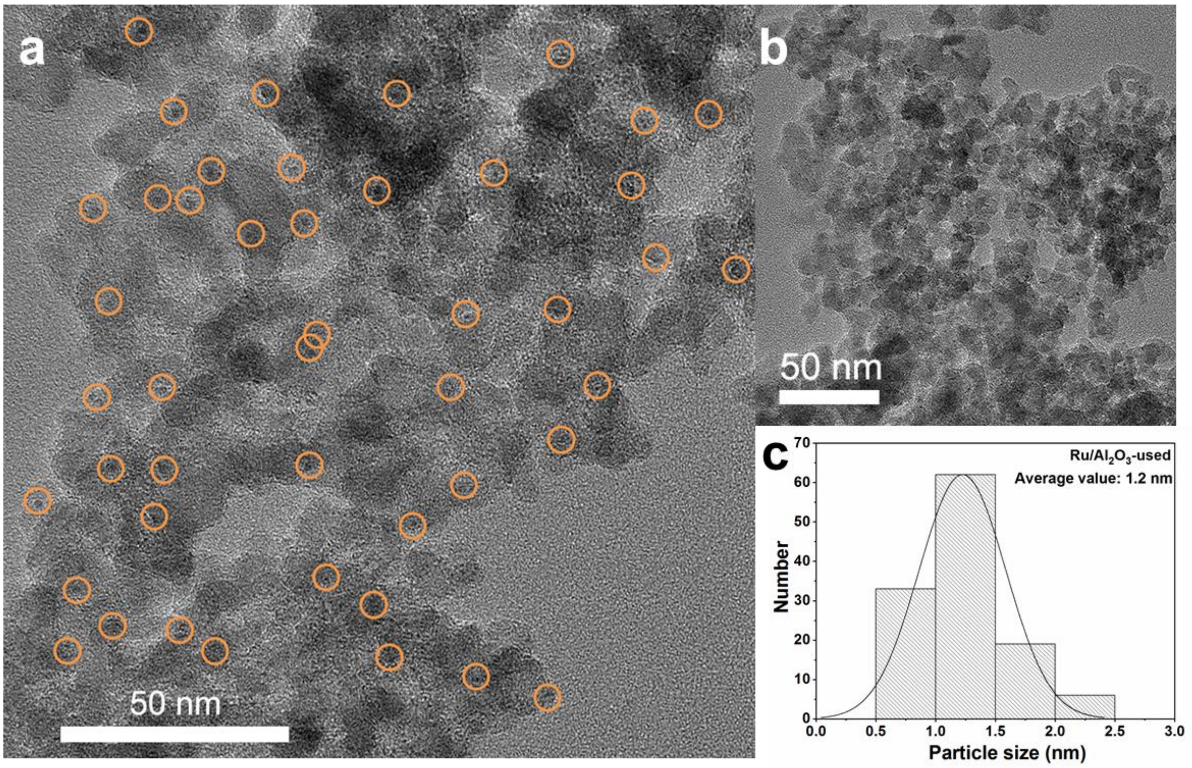

Supplementary Fig. 14 | HRTEM images of the used Ru/Al_2O_3. (a, b) HRTEM images of the Ru/Al_2O_3-used catalyst; (c) the particle size distribution of Ru species for Ru/Al_2O_3-used catalyst.

Supplementary Note 11: The average particle size of Ru clusters on Ru/Al_2O_3-used catalyst was 1.2 nm.

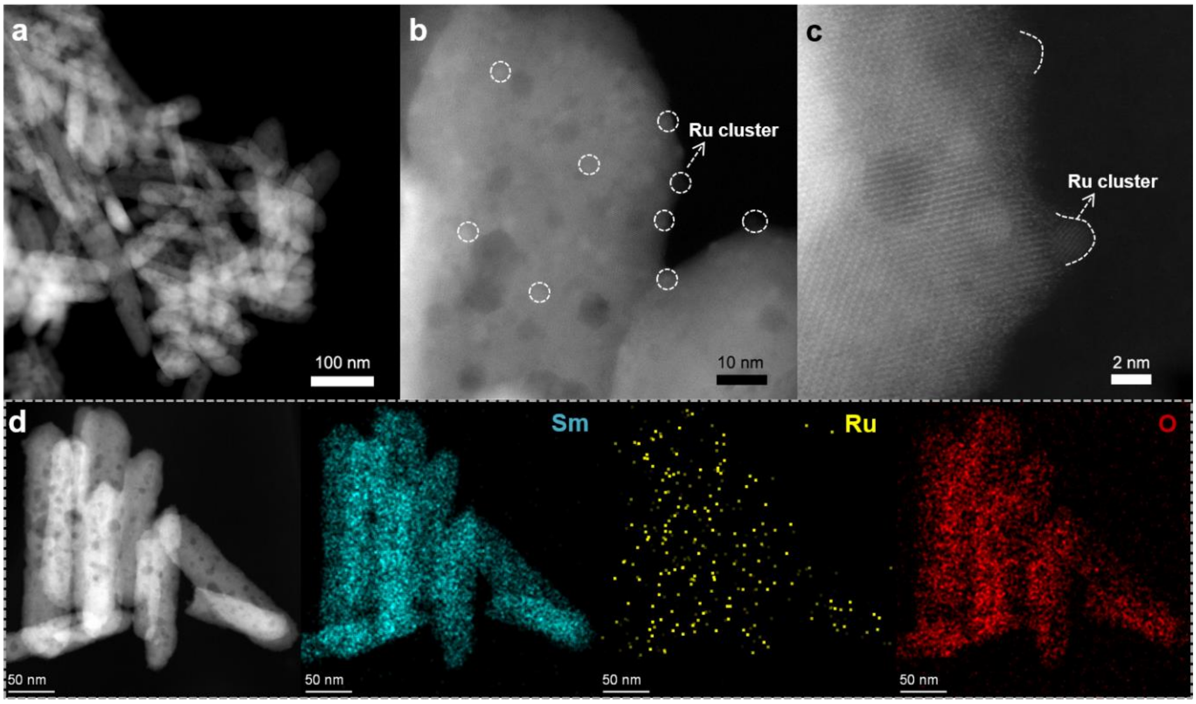

Supplementary Fig. 15 | HAADF-STEM images of the used Ru/Sm_2O_3. (a−c) The aberration-corrected HAADF-STEM images of the used Ru/Sm_2O_3; (d) EDS elemental mapping results of the used Ru/Sm_2O_3.

Supplementary Note 12: The Ru species was uniform distribution on the Sm_2O_3 support.

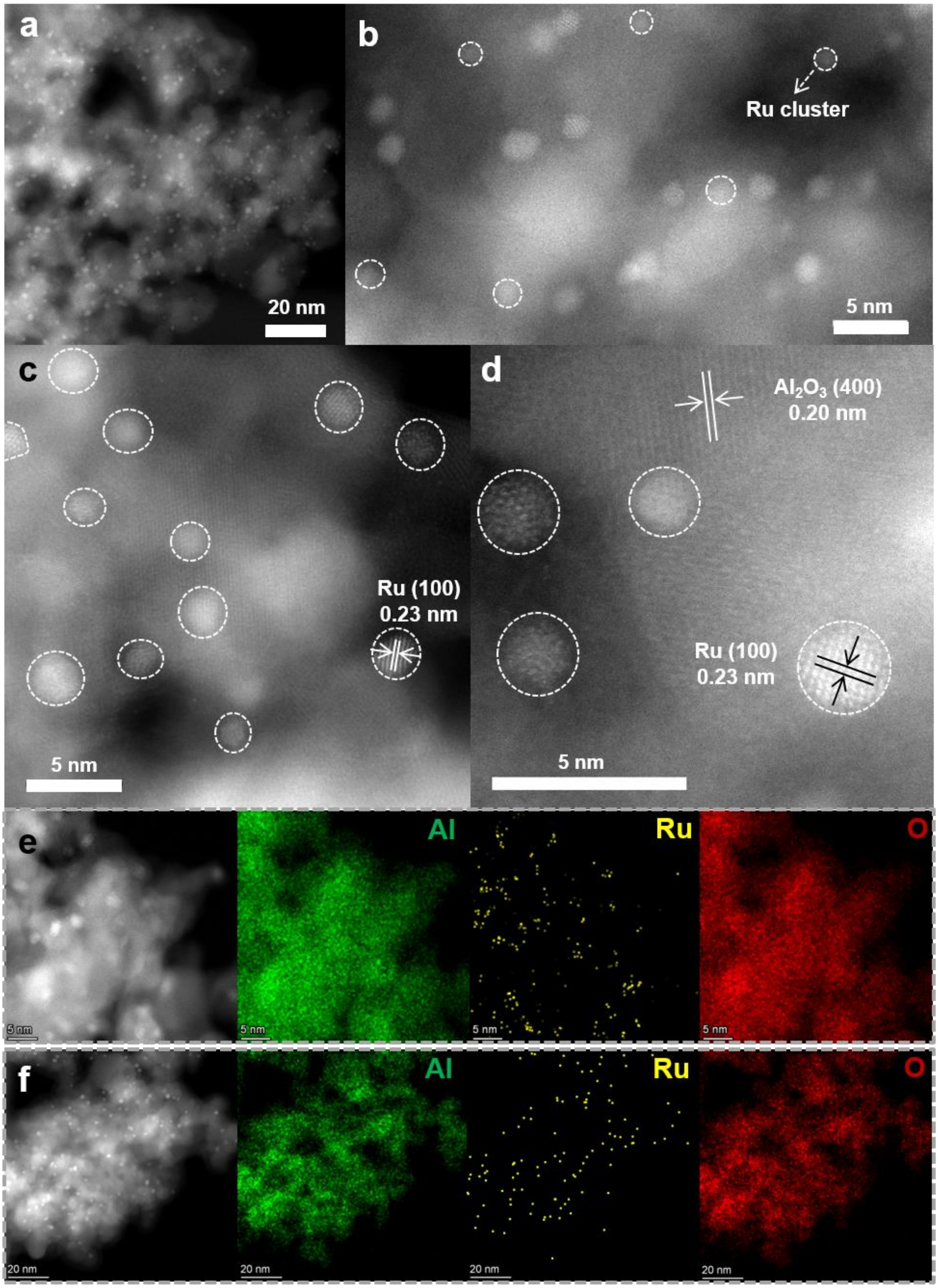

Supplementary Fig. 16 | HAADF-STEM images of the used Ru/Al_2O_3. (a–d) The aberration-corrected HAADF-STEM images of Ru/Al_2O_3; (e, f) EDS elemental mapping results of Ru/Al_2O_3.

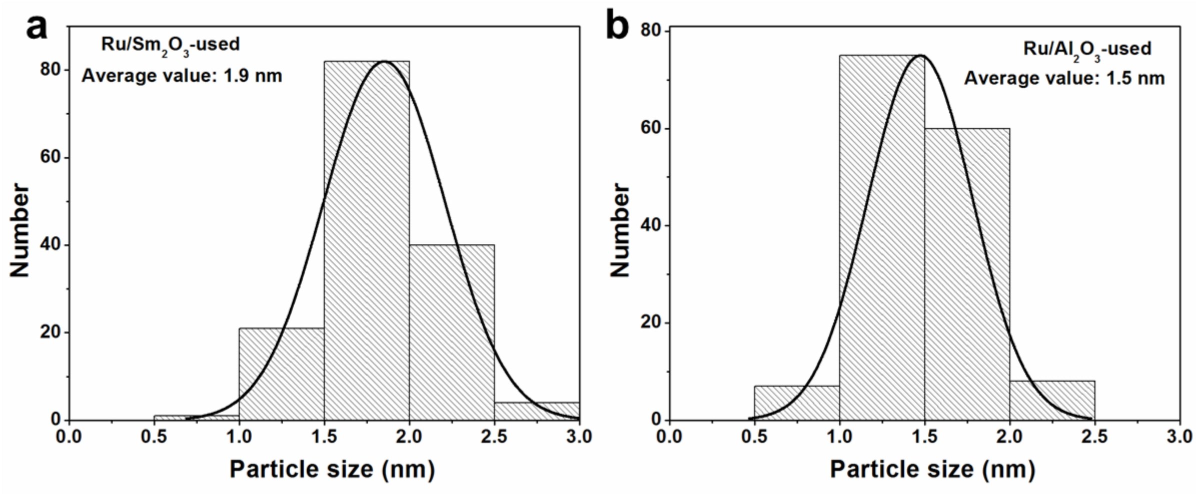

Supplementary Fig. 17 | The particle size distributions of Ru species in the aberration-corrected HAADF-STEM images. (a) Ru/Sm_2O_3-used, (b) Ru/Al_2O_3-used.

Supplementary Note 13: According to the results of particle size distributions, the Ru species on both Sm_2O_3 and Al_2O_3 existed as nanoclusters < 2 nm.

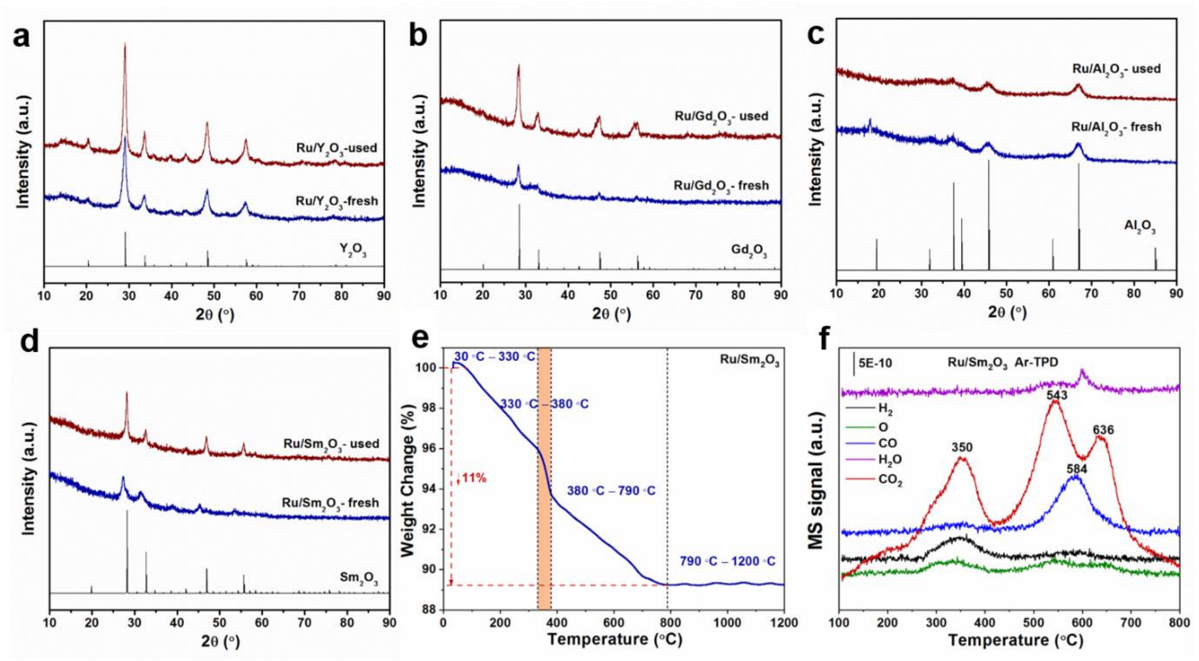

Supplementary Fig. 18 | XRD patterns and the temperature-programmed desorption (TPD) analysis. XRD patterns of (a) Ru/Y_2O_3; (b) Ru/Gd_2O_3; (c) Ru/Al_2O_3; (d) Ru/Sm_2O_3; (e) TGA curves of Ru/Sm_2O_3 catalyst in N_2 flow; (f) the TPD profiles using Ar as the carrier gas for the Ru/Sm_2O_3 catalyst.

Supplementary Note 14: The XRD results before and after the reaction of all prepared Ru-based catalysts showed mainly the support information without the appearance of diffraction peaks associated with Ru species. For the Ru/Sm_2O_3-fresh sample, the deviation of diffraction peak related to the existence of OH and carbonate through the results of TG and TG-MS.

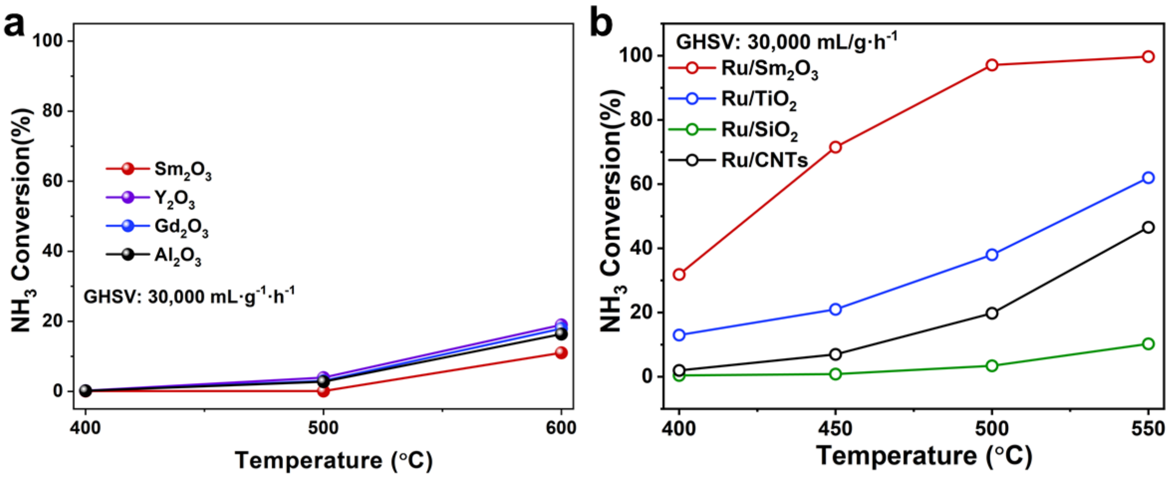

Supplementary Fig. 19 | Temperature-dependent activities of the catalysts and oxides, GHSV = 30,000 mL·g^{-1}·h^{-1}. (a) Sm_2O_3, Y_2O_3, Gd_2O_3 and Al_2O_3; (b) Ru/Sm_2O_3, Ru/TiO_2, Ru/SiO_2 and Ru/CNTs

Supplementary Note 15: Comparing the catalytic activity of the supports with that of the Ru-loaded catalysts, it could be clearly observed that the catalytic activity for ammonia decomposition for these metal oxides themselves was very low. Besides, the catalysts that supported on TiO_2, SiO_2, CNTs were prepared, which showed low NH_3 conversion during the test process.

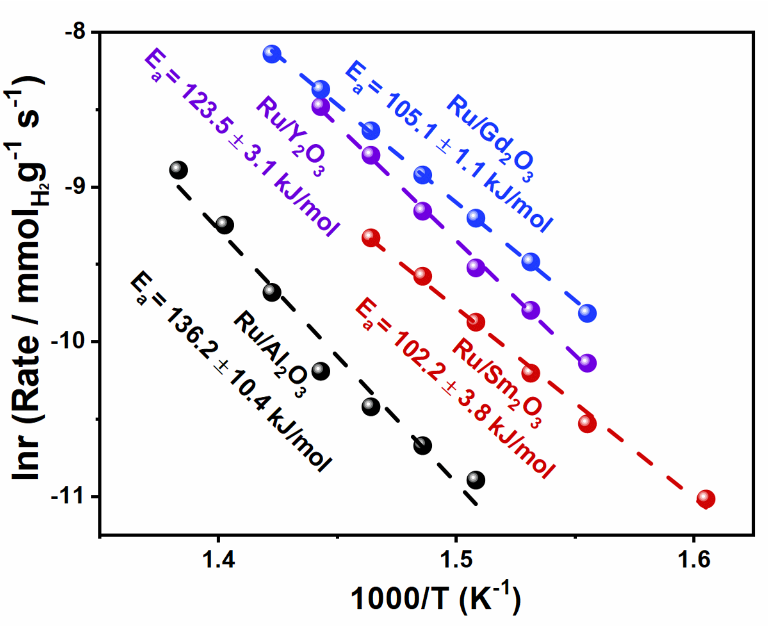

Supplementary Fig. 20 | Arrhenius plots for the catalysts. (Ru/Sm_2O_3, Ru/Y_2O_3, Ru/Gd_2O_3 and Ru/Al_2O_3) in the kinetic range.

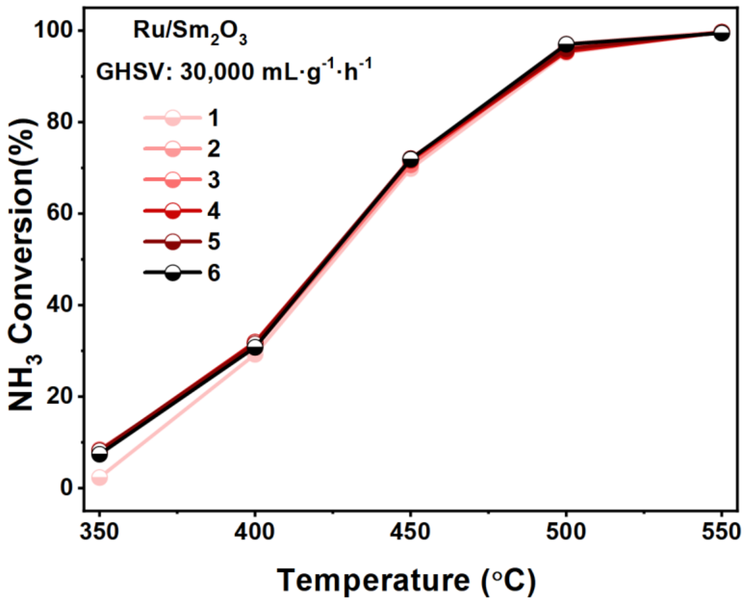

Supplementary Fig. 21 | Cyclic stability test (GHSV = 30,000 mL·g^{-1}·h^{-1}) of Ru/Sm_2O_3 catalyst for ammonia decomposition.

Supplementary Note 16: After six cycles of activity tests, the NH_3 conversion of Ru/Sm_2O_3 catalyst remained constant, indicating that it had very good stability.

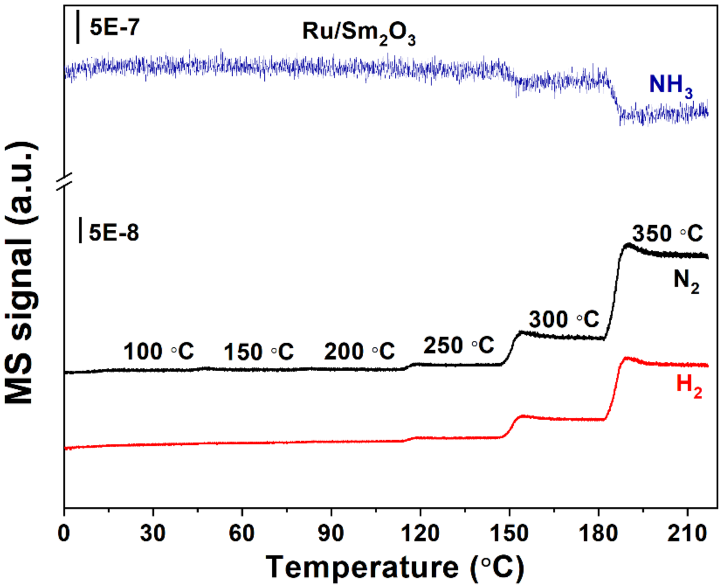

Supplementary Fig. 22 | Ru/Sm_2O_3 catalyst for ammonia decomposition reaction at low temperature.

Supplementary Note 17: In the test result of Ru/Sm_2O_3, the catalyst had started catalyzing the reaction at a low temperature of 200 °C and had obvious catalytic activity at 250 °C, showing extraordinary catalytic performance.

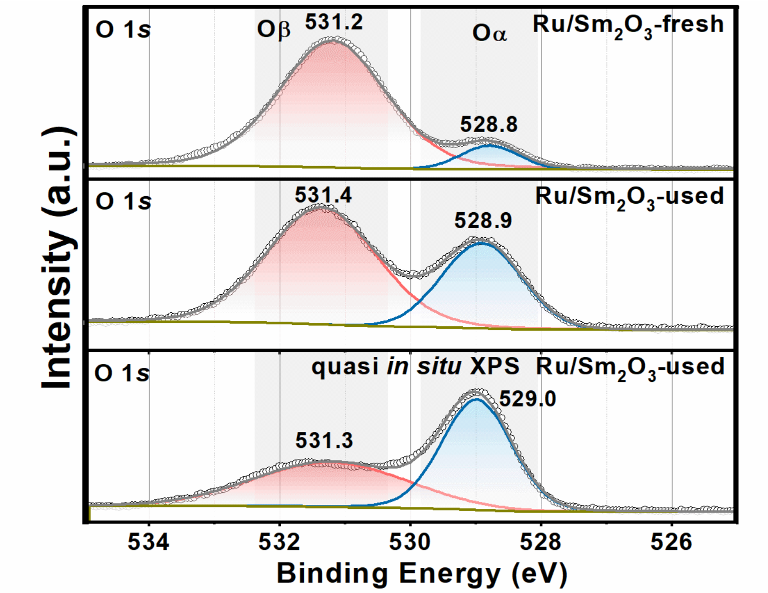

Supplementary Fig. 23 | O 1*s* XPS results of the fresh and used Ru/Sm_2O_3 catalysts.

Supplementary Note 18: For the Ru/Sm_2O_3 catalyst, Ru 3*d* spectrum (Fig. 3a) showed Ru^{4+} (281.5 eV) for the fresh sample, and Ru^{4+} together with Ru^0 (280.9 and 280.5 eV) for the used sample and sample measured at quasi *in situ* condition[4,5]. In the Sm 3*d* spectra (Fig. 3b), Sm^{3+} was the only detected species. The O 1*s* spectrum mainly exhibited two peaks (Supplementary Fig. 18). The peaks at 528.8, 528.9 and 529.0 eV were assigned to the lattice oxygen denoted as O_α, and the peaks at 531.2, 531.3 and 531.4veV was attributed to the adsorbed oxygen, oxygen in hydroxyl-like groups and surface oxygen bounded with Ru atoms of the catalyst denoted as O_β[4,6,7]. After the reaction, the peak of O_β became weaker which might be caused by the reduction of surface oxygen bounded with Ru atoms and the desorption of surface oxygen under high temperature vacuum environment.

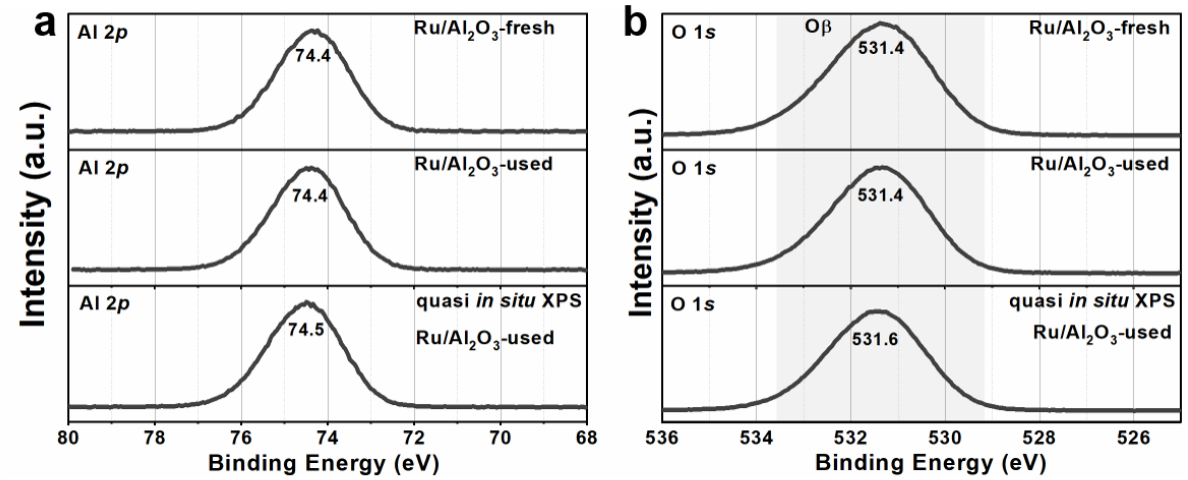

Supplementary Fig. 24 | XPS results of the fresh and used Ru/Al_2O_3 catalysts. (a) Al 2*p*; (b) O 1*s*.

Supplementary Note 19: For the Ru/Al_2O_3 catalyst, Ru 3*d* spectrum (Fig. 3c) showed mainly Ru^{4+} (281.3 eV) for the fresh and used samples. and Ru^{4+} together with Ru^0 (281.0 eV) for the sample measured at quasi *in situ* condition. For the Al 2*p* spectra (Supplementary Fig. 19a), the Al^{3+} was the only species detected. For the O 1*s* spectra (Supplementary Fig. 19b), mainly the peak of O_β was observed, and there was no obvious change for the fresh and used samples.

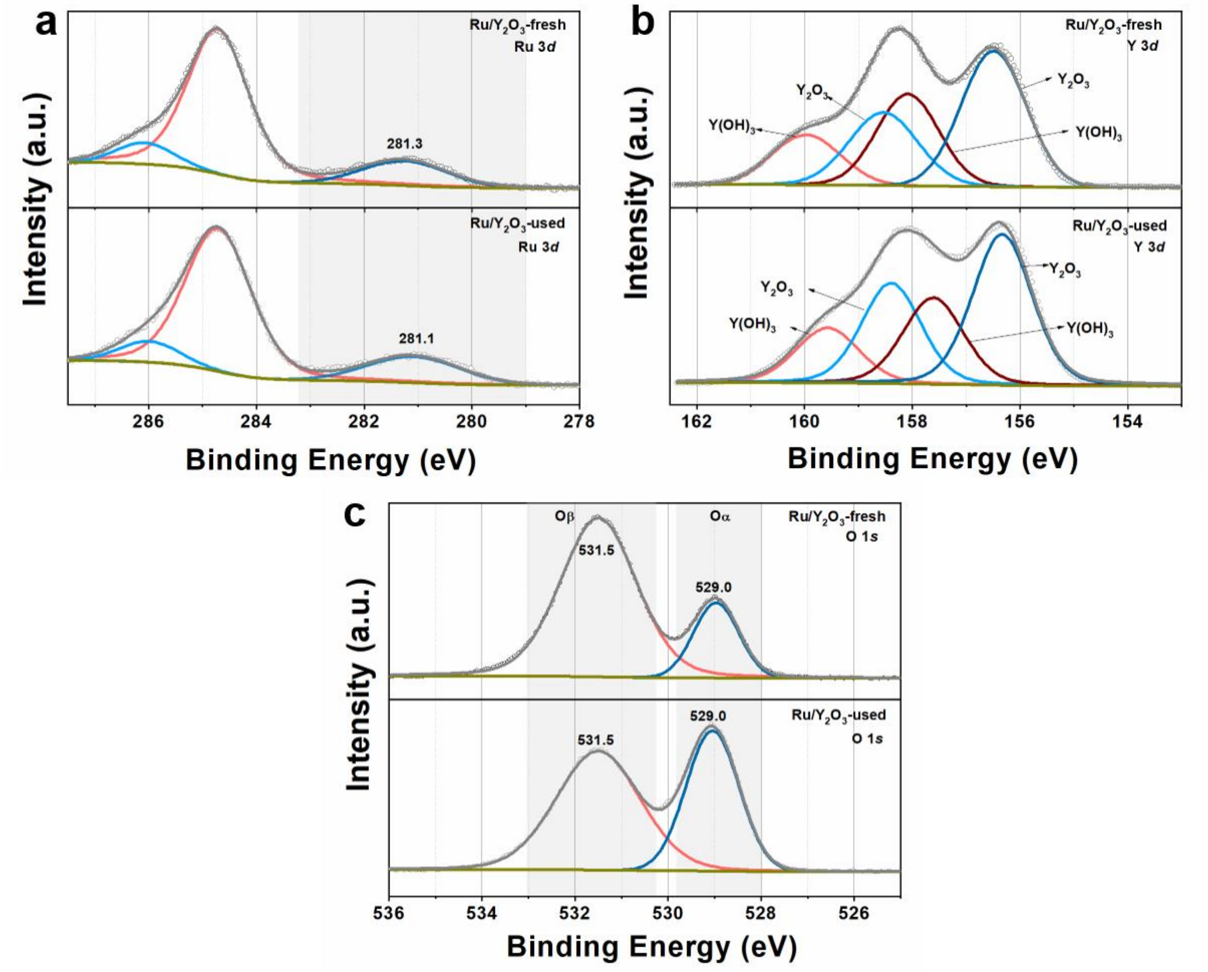

Supplementary Fig. 25 | XPS results of the fresh and used Ru/Y_2O_3 catalysts. (a) Ru 3*d*; (b) Y 3*d*; (c) O 1*s*.

Supplementary Note 20: For the Ru/Y_2O_3 catalyst, Ru 3*d* spectrum (Supplementary Fig. 20a) showed Ru^{4+} (281.3 eV) for the fresh sample, and Ru^{4+} together with Ru^0 (281.1 eV) for the used sample. For the Y 3*d* spectra (Supplementary Fig. 20b), the Y^{3+} was the only species detected. After the reaction, the peak of O_β become weaker which might be caused by the reduction of surface oxygen bounded with Ru atoms and the desorption of surface oxygen under high temperatures vacuum environment (Supplementary Fig. 20c).

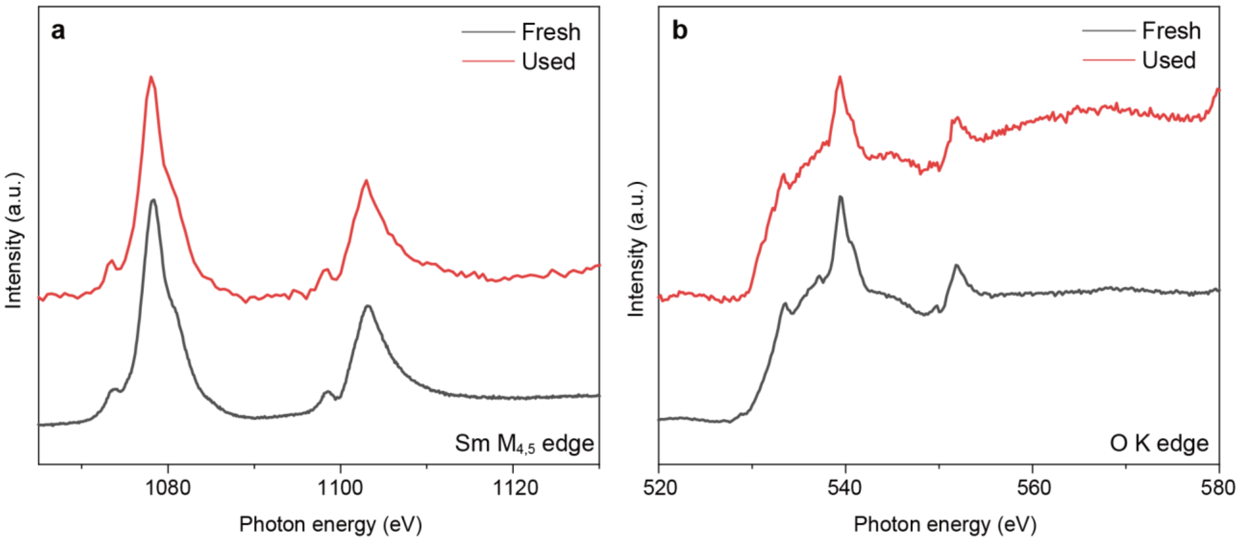

Supplementary Fig. 26 | NEXAFS of the fresh and used Ru/Sm_2O_3 catalysts. (a) Sm $M_{4,5}$ edge; (b) O K edge.

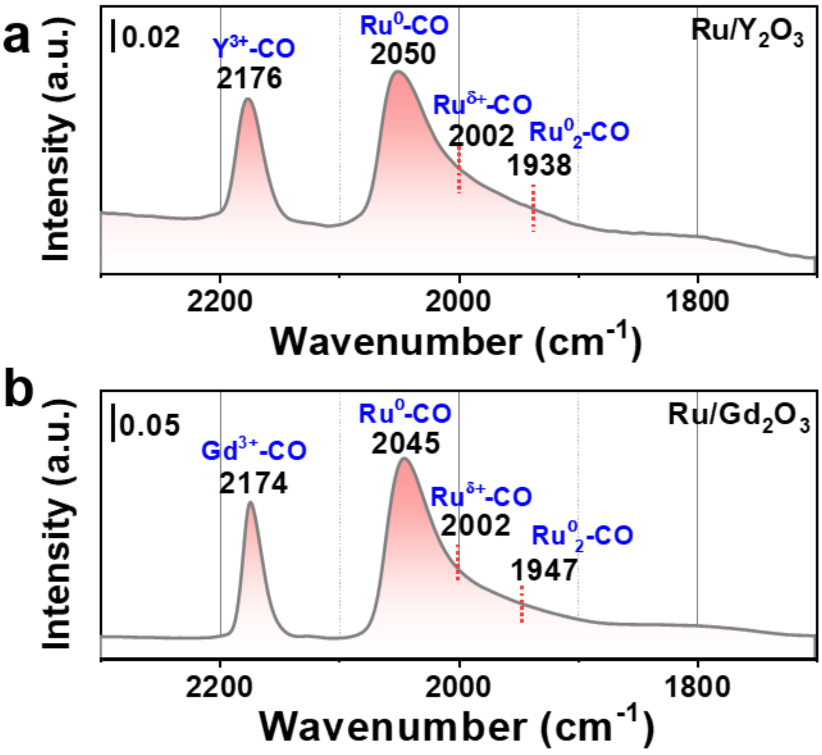

Supplementary Fig. 27 | The *in situ* infrared spectroscopy in the transmission mode of samples at 130K. (a) Ru/Y_2O_3 and (b) Ru/Gd_2O_3.

Supplementary Note 21: The main peak position of Ru/Sm_2O_3, Ru/Y_2O_3 and Ru/Gd_2O_3 was concentrated at 2041, 2050 and 2045 cm^{-1}, respectively. This position was considered as the adsorption of CO on Ru^0 species[8–10]. In comparison, the main peak position of Ru/Al_2O_3 was concentrated at 2064 cm^{-1}, which was considered as the CO adsorption on $Ru^{\delta+}$ species. The shoulder peaks also contained other forms of CO adsorption on $Ru^{\delta+}$ and Ru^0 species, indicating the presence of Ru species as clusters.

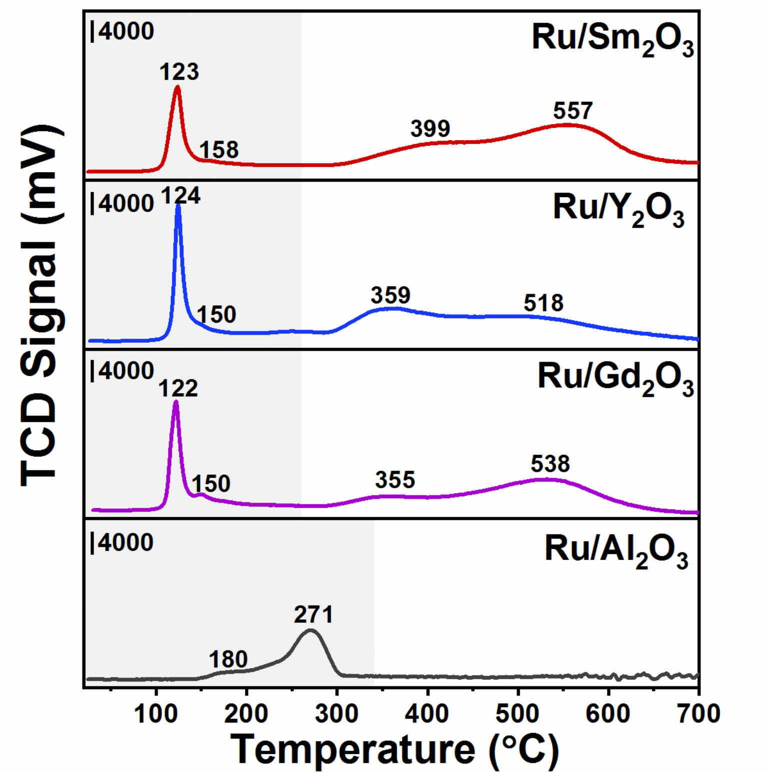

Supplementary Fig. 28 | H_2-TPR profiles of the fresh catalysts (Ru/Sm_2O_3, Ru/Y_2O_3, Ru/Gd_2O_3 and Ru/Al_2O_3).

Supplementary Note 22: To explore the redox property of the catalysts, the temperature programmed reduction by hydrogen (H_2-TPR) measurement after pretreatment at 500 °C in air was carried out. For Ru/Sm_2O_3, Ru/Y_2O_3 and Ru/Gd_2O_3 catalysts, the reduction peaks between 100−160 °C were considered to be the Ru species reduction. The H_2 consumption between 350−400 °C was considered to be the hydrogenation reaction that occurred to produce CH_4, and the higher temperature peaks (500−560 °C) mainly related to the reduction of surface carbonate species (as seen in Supplementary Fig. 29). Therefore, we focused on the reduction of Ru species in the lower temperature regions. For Ru/Sm_2O_3, Ru/Y_2O_3 and Ru/Gd_2O_3 catalysts, there was mainly one sharp peak at ~123 °C. It was similar to the previously reported reduction peaks in Ru/CeO_2 catalyst[4], which could be attributed to the reduction of highly dispersed single-atom Ru species into nanoclusters. Unlike these Ru-based rare earth oxide catalysts, the reduction peaks of Ru species in Ru/Al_2O_3 catalyst were at higher temperatures and showed a broad peak pattern. These results indicated that the Ru species on γ-Al_2O_3 did not form a uniformly dispersed state as that in Ru/RE_2O_3 after pretreatment.

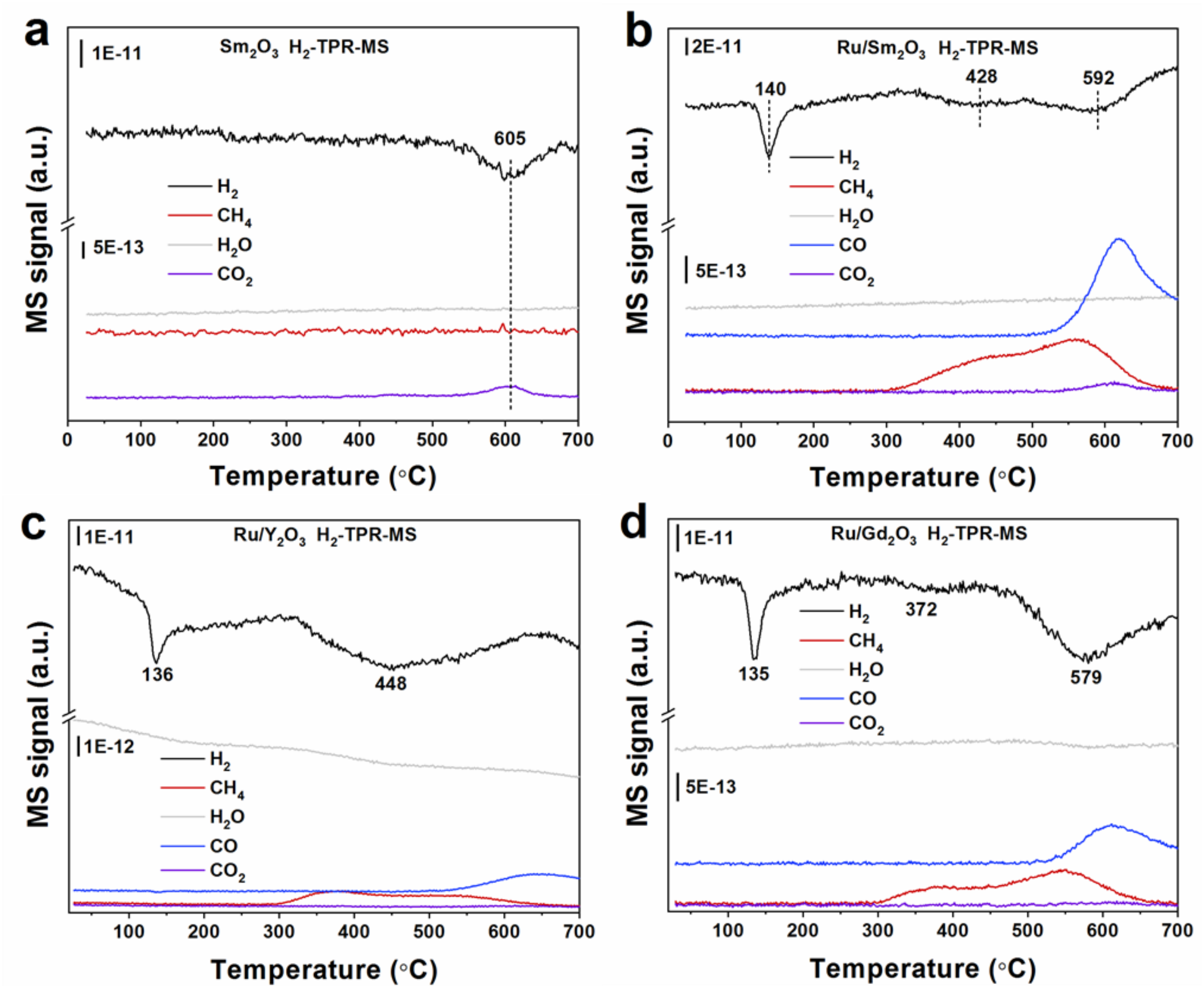

Supplementary Fig. 29 | H_2-TPR profile of the catalysts with a mass spectrometer: (a) Sm_2O_3; (b) Ru/Sm_2O_3; (c) Ru/Y_2O_3; (d) Ru/Gd_2O_3.

Supplementary Note 23: Supplementary Fig. 29 showed the signals of each component detected by mass spectrometer during the heating process of each sample. The results showed that pure Sm_2O_3 support was not easily reduced. The H_2 consumption at 605 °C related to the reduction of surface carbonate species. And according to the results of mass spectrometry, the peaks above 300 °C were generated by methanation and carbonate reduction. Therefore, the reduction peaks of Ru species were mainly concentrated before 200 °C.

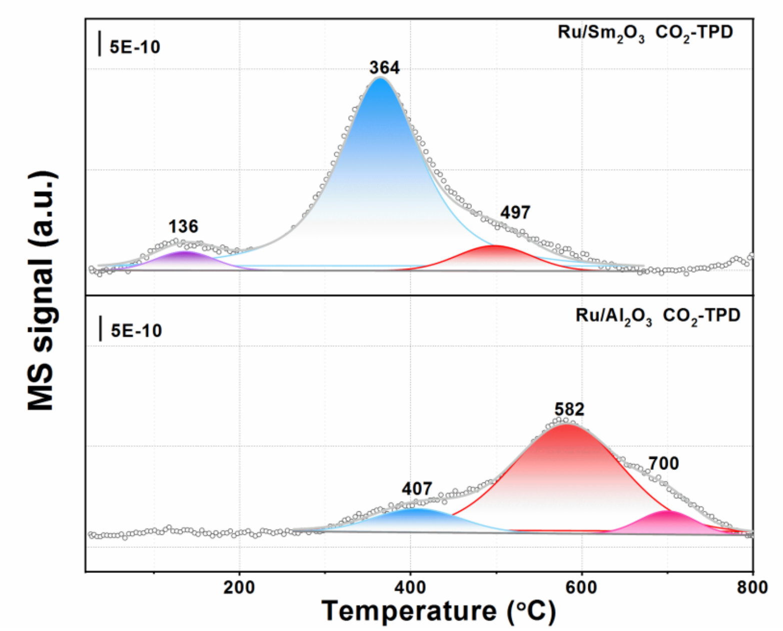

Supplementary Fig. 30 | CO_2-TPD profiles of the Ru/Sm_2O_3 and Ru/Al_2O_3 catalyst.

Supplementary Note 24: According to the electron donor-acceptor theory, the stronger surface basicity was more conducive to the electron transfer from the support to Ru species, and further facilitated the dissociative adsorption of N species. Both the medium and strong base site (> 130 °C) were considered formed by surface oxygen ion, which was conducive to providing electrons to Ru species. And medium base sites were thought to be more favourable for ammonia decomposition[11]. The CO_2-TPD result showed CO_2 desorption mainly concentrated at 364 °C on Ru/Sm_2O_3, which was mainly medium base sites. The desorption peak of Ru/Al_2O_3 was mainly at a higher temperature of 582 °C, indicating that there are strong base sites. According to the peak area, the CO_2 desorption of Ru/Sm_2O_3 was 1.2 times more than that of Ru/Al_2O_3, indicating that Ru/Sm_2O_3 had more effective surface basicity and could activate molecules more favourably.

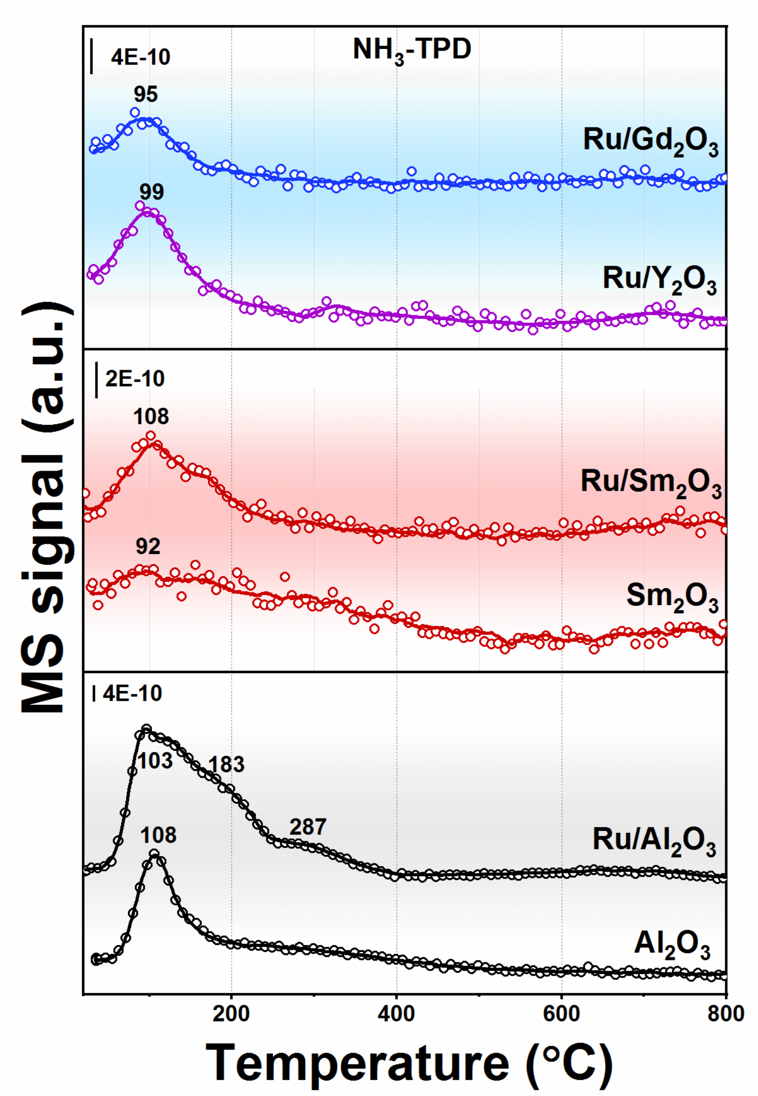

Supplementary Fig. 31 | The NH_3-TPD results. The NH_2 signal of NH_3-TPD for the catalysts.

Supplementary Note 25: In order to observe the desorption of NH_3 (m/z=17) more realistically, we analyzed the NH_2 (m/z=16) signal to exclude the interference of OH (m/z=17). Comparing the NH_2 desorption signals of Ru/RE_2O_3 and Ru/Al_2O_3, it could be clearly observed that Ru/Al_2O_3 has significantly more NH_3 desorption than Ru/RE_2O_3 and the desorption temperature was also higher than of Ru/RE_2O_3. These results indicated that Ru/Al_2O_3 surface had more adsorption sites and stronger adsorption, but it could not be considered that this adsorption was favorable for NH_3 dissociation. In addition, for the oxide supports, the same NH_2 desorption was observed as for Ru/RE_2O_3, indicating that the oxide supports were mainly used to adsorb NH_3.

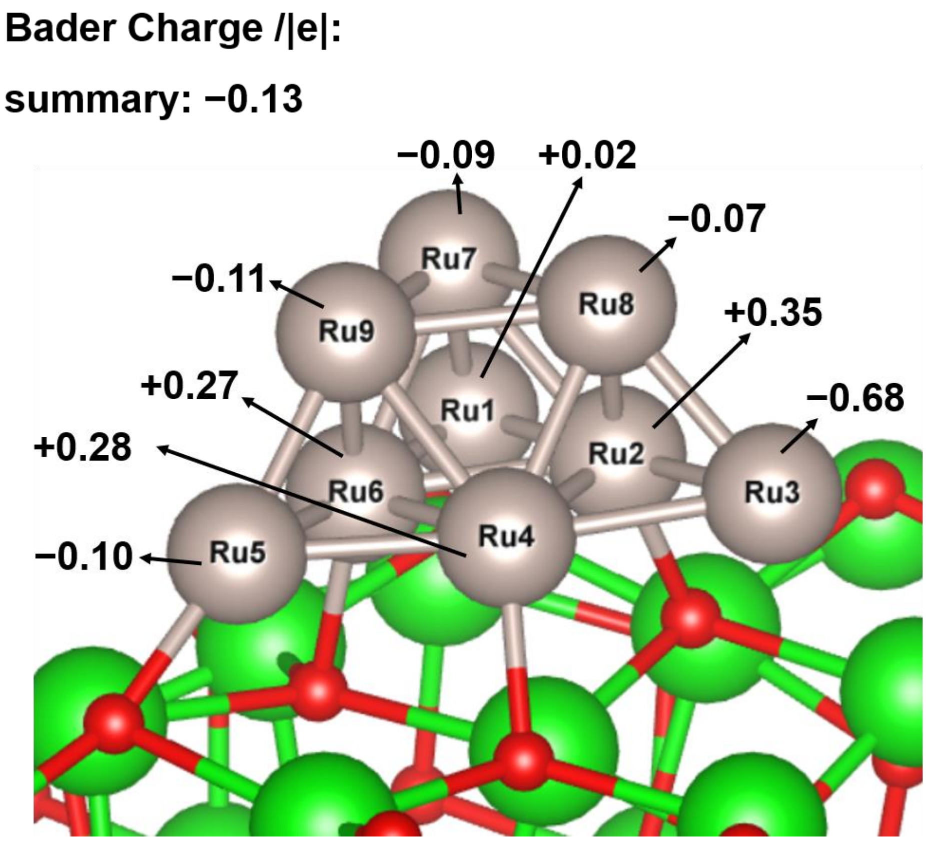

Supplementary Fig. 32 | The Bader charge of every Ru atoms in the Ru_9 cluster on the Sm_2O_3 (111) surface.

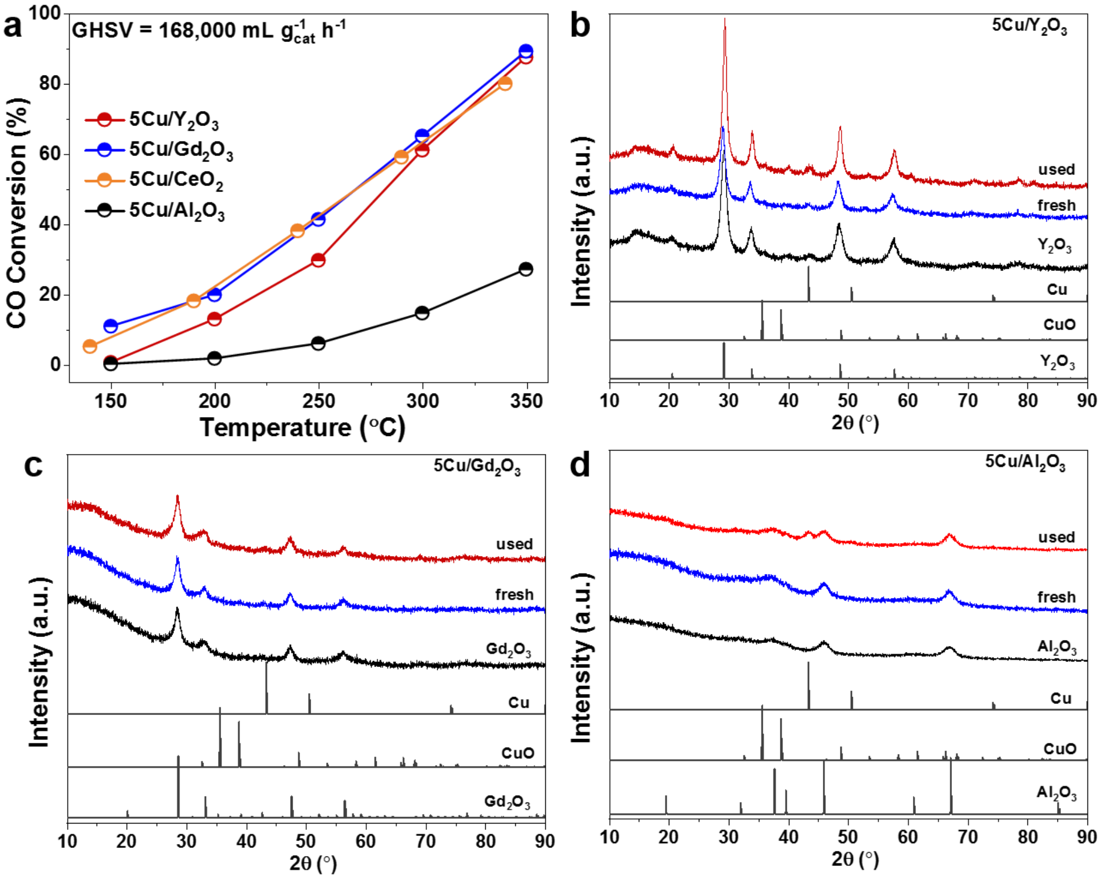

Supplementary Fig. 33 | Catalytic performance and XRD patterns of Cu/Y_2O_3, Cu/Gd_2O_3, Cu/CeO_2 and Cu/Al_2O_3 for WGS reaction. (a) Temperature-dependent activity test of Cu/Y_2O_3, Cu/Gd_2O_3, Cu/CeO_2 and Cu/Al_2O_3 for the WGS reaction; (b) XRD patterns of the fresh and used Cu/Y_2O_3 catalyst; (c) XRD patterns of the fresh and used Cu/Gd_2O_3 catalyst; (d) XRD patterns of the fresh and used Cu/Al_2O_3 catalyst.

Supplementary Note 26: By comparing the activity results of Cu/RE_2O_3 and Cu/Al_2O_3 catalyzing the WGS reaction, it could be found that the activity of Cu/RE_2O_3 was significantly higher than that of Cu/Al_2O_3. The catalytic performance test results verify the advantages of rare earth oxides with intrinsic surface O_V. The XRD results showed that these catalysts mainly contained crystalline phases of Y_2O_3, Gd_2O_3 and Al_2O_3. Meanwhile, all catalysts exhibited metallic Cu diffraction peaks after the reaction, probably due to the agglomeration of Cu species after high-temperature reaction in a reducing atmosphere.

Supplementary Tables

Supplementary Table 1 The adsorption energy of molecules (NH_3, H_2O and O_2) on the different sites.

Samples	Adsorption energy for NH_3 (eV)	Adsorption energy for H_2O (eV)	Adsorption energy for O_2 (eV)
Sm_2O_3 (111)	−0.44	-1.52	-1.24
Sm_2O_3(111)-non-vacancy site	-0.27	-0.28	-0.01
Sm_2O_3(110)	-0.36	-1.03	-0.71
Sm_2O_3(100)	-0.98	-2.41	-2.31
Gd_2O_3 (111)	−0.45	–	–
CeO_2 (111)	−0.56	–	–
CeO_2(111)-O_v	-0.58	-2.71	-2.51
Al_2O_3 (111)	−1.74	–	–
Ru_9/Sm_2O_3	−0.79	–	–
Ru_9/Al_2O_3	−1.88	–	–

Supplementary Table 2 The Ru contents and S_{BET} of the Ru-based catalysts.

Catalysts	Ru loading (%)[a]	S_{BET} ($m^2 \cdot g^{-1}$) [b]
Ru/Sm_2O_3-fresh	1.03	27.38
Ru/Sm_2O_3-used	——	27.07
Ru/Y_2O_3-fresh	1.06	54.24
Ru/Y_2O_3-used	——	59.03
Ru/Gd_2O_3-fresh	0.82	45.77
Ru/Gd_2O_3-used	——	39.53
Ru/Al_2O_3-fresh	0.74	143.12
Ru/Al_2O_3-used	——	150.68

[a] Determined by ICP-MS analysis.

[b] Determined with N_2 adsorption.

Supplementary Table 3 Comparison of catalytic performances for ammonia decomposition reaction over various catalysts at 450 °C.

Catalysts	Metal loading (wt.%)	GHSV (NH_3 mL· g_{cat}^{-1}·h^{-1})	Yield (mmolH_2· g_{cat}^{-1}·min^{-1})	Yield (mmolH_2· G_{metal}^{-1}·min^{-1})	Reference
Ru/Sm_2O_3	1.03	300,000	138.2	13417	This work
Ru/Sm_2O_3	1.03	30,000	24.0	2325	This work
Ru/CeO_2	1.00	300,000	115.7	11570	This work
Ru/CeO_2	1.00	30,000	24.1	2410	This work
Ru/Y_2O_3	1.06	300,000	124.9	11783	This work
Ru/Y_2O_3	1.06	30,000	20.4	1926	This work
Ru/Gd_2O_3	0.82	300,000	103.4	12610	This work
Ru/Gd_2O_3	0.82	30,000	21.4	2613	This work
Ru/Al_2O_3	0.74	300,000	41.6	5622	This work
Ru/Al_2O_3	0.74	30,000	4.3	574	This work
Ru/Sm_2O_3-p	3.83	30,000	25.9	683	12
Ru/Y_2O_3-p	5.00	30,000	25.7	513	13
K-Ru/MgO	3.50	36,000	32.1	914	14
K–Ru/CNTs	4.80	30,000	29.9	622	15
Ru/MgO–CNTs	4.85	60,000	18.4	379	16
Ru/c-MgO	4.70	30,000	24.7	526	17
Ru/$CaAlO_x$-w	3.50	30,000	6.1	175	18
Ru-Ni/CeO_2	0.35	15,000	6.2	1767	19
Ru/MgO	1.70	22,000	18.7	1098	20
Ru/$LaCeO_x$	1.80	6,000	6.1	341	11
Ru/CNF	3.20	6,500	5.3	164	21
Ru-Cs-Mg/MIL-101	3.10	15,000	15.3	495	22
Ru/Al_2O_3	4.00	12,000	12.0	301	23
Ru-K/ZrO_2	4.40	150,000	17.1	388	24
Ru-K/CNT	4.50	150,000	26.0	577	
Ru/SiO_2	10.00	30,000	11.2	112	25
Co/Y_2O_3	10.00	6,000	1.9	19	26
Ni/Y_2O_3 (500°C)	10.00	6,000	3.3	33	27
$Co_{0.7}Sm_{0.3}O_x$ (500°C)	-	-	97.2	-	28
Co/La-MgO(5) (550°C)	20.00	124,000	91.0	455	
Ni/La-MgO(5) (550°C)	20.00	124,000	86.0	430	29
Fe/La-MgO(5) (550°C)	20.00	124,000	60.0	300	
Fe-CNFs/mica (600°C)	-	6,500	7	-	30

Supplementary Table 4 The atomic% results of Ru and RE (Sm and Y) in XPS.

Samples	Atomic%	
	Ru	RE (Sm and Y)
Ru/Sm_2O_3-fresh	1.0	13.9
Ru/Sm_2O_3-used	0.9	21.1
Ru/Y_2O_3-fresh	0.4	26.6
Ru/Y_2O_3-used	0.7	34.3

Supplementary References

1. Farmer, J. A., Campbell, C. T. Ceria maintains smaller metal catalyst particles by strong metal-support bonding. *Science* **329**, 933−936 (2010).
2. Esch, F. et al. Electron localization determines defect formation on ceria substrates. *Science*, **309**, 752−755 (2005).
3. Zhang, S. et al. Solid frustrated-Lewis-pair catalysts constructed by regulations on surface defects of porous nanorods of CeO_2. *Nat. Commun.* **8,** 15266 (2017).
4. Hu, X.-C. et al. Ceria-supported ruthenium clusters transforming from isolated single atoms for hydrogen production via decomposition of ammonia. *Appl. Catal. B. Environ.* **268,** 18424 (2020).
5. Kim, K. S. and Winograd, N. X-ray photoelectron spectroscopic studies of ruthenium-oxygen surfaces. *J. Catal.* **35,** 66−72 (1974).
6. Jiang, F. et al. Insights into the influence of CeO_2 crystal facet on CO_2 hydrogenation to methanol over Pd/CeO_2 catalysts. *ACS Catal.* **10,** 1493−11509 (2020).
7. Huang, H., Dai, Q. and Wang, X. Morphology effect of Ru/CeO_2 catalysts for the catalytic combustion of chlorobenzene. *Appl. Catal. B. Environ.* **158−159,** 96−105 (2014).
8. Hadjiivanov, K. et al. FTIR study of CO interaction with Ru/TiO_2 catalysts. *J. Catal.* **176,** 415−425 (1998).
9. Chin, S. Y., Williams C. T. and Amiridis M. D. FTIR studies of CO adsorption on Al_2O_3- and SiO_2-supported Ru catalysts. *J. Phys. Chem. B.* **110,** 871−882 (2006).
10. Luo, W. et al. High performing and stable supported nano-alloys for the catalytic hydrogenation of levulinic acid to γ-valerolactone. *Nat. Commun.* **6,** 6540 (2015).
11. Le, T. A. et al. Ru-supported lanthania-ceria composite as an efficient catalyst for CO_x-free H_2 production from ammonia decomposition. *Appl. Catal. B. Environ.* **285,** 119831 (2021).
12. Zhang, X. et al. Metal–support interaction-modulated catalytic activity of Ru nanoparticles on Sm_2O_3 for efficient ammonia decomposition. *Catal. Sci. Technol.* 11, 2915−2923 (2021).
13. Feng, J. et al. Highly dispersed ruthenium nanoparticles on Y_2O_3 as superior catalyst for ammonia decomposition. *ChemCatChem,* **13,** 1552−1558 (2021).
14. Ju, X. et al. Mesoporous Ru/MgO prepared by a deposition-precipitation method as highly active catalyst for producing CO_x-free hydrogen from ammonia decomposition. *Appl. Catal. B. Environ.* **211,** 167−175 (2017).

15. Yin, S. F. et al. Carbon nanotubes-supported Ru catalyst for the generation of CO_x-free hydrogen from ammonia. *Catal. Today*. **93−95,** 27−38 (2004).

16. Yin, S. F. et al. Magnesia–carbon nanotubes (MgO–CNTs) nanocomposite: novel support of Ru catalyst for the generation of CO_x-free hydrogen from ammonia. *Catal. letters* **96,** 113−116 (2004).

17. Ju, X. et al. Highly efficient Ru/MgO catalyst with surface−enriched basic sites for production of hydrogen from ammonia decomposition. *ChemCatChem,* **11,** 4161−4170 (2019).

18. Zhao, J. et al. Metal–support interactions on Ru/$CaAlO_x$ catalysts derived from structural reconstruction of Ca–Al layered double hydroxides for ammonia decomposition. *Chem. Commun.* **55,** 14410 (2019).

19. Lucentini, I. et al. Catalytic ammonia decomposition over Ni-Ru supported on CeO_2 for hydrogen production: Effect of metal loading and kinetic analysis. *Appl. Catal. B. Environ.* **286,** 119896 (2021).

20. Hu, X. et al., Hydrogen production via catalytic decomposition of NH_3 using promoted MgO-supported ruthenium catalysts. *Sci. China Chem.* **62**, 1625−1633 (2019).

21. Duan, X. et al. Carbon nanofiber-supported Ru catalysts for hydrogen evolution by ammonia decomposition. *Chin. J. Catal.* **31,** 979−986 (2010).

22. Li, J. et al. Sub-nm ruthenium cluster as an efficient and robust catalyst for decomposition and synthesis of ammonia: Break the "size shackles". *Nano Research* **11,** 4774−4785 (2018).

23. Karim, A. M. et al. Correlating particle size and shape of supported Ru/γ-Al_2O_3Catalysts with NH_3 decomposition activity. *J. Am. Chem. Soc.* **131,** 12230−12239 (2009).

24. Yin, S. et al. Investigation on the catalysis of CO_x-free hydrogen generation from ammonia. *J. Catal.* **224,** 384−396 (2004).

25. Choudhary, T. V. et al. Catalytic ammonia decomposition: CO_x-free hydrogen production for fuel cell applications. *Catal. Lett.* **72,** 197–201 (2001).

26. Huang, C. et al. $Ce_{0.6}Zr_{0.3}Y_{0.1}O_2$ solid solutions-supported Ni–Co bimetal nanocatalysts for NH_3 decomposition. *Appl. Surf. Sci.* **478**, 708 (2019).

27. Okura, K. et al. Ammonia Decomposition over Nickel Catalysts Supported on Rare-Earth Oxides for the On-Site Generation of Hydrogen. *ChemCatChem*, **8**, 2988 (2016).

28. Wu, C.-P. et al. $Co_aSm_bO_x$ Catalyst with Excellent Catalytic Performance for NH_3 Decomposition. *Chin. J. Chem.* **39**, 2359 (2021).

29. Hu, X.-C. Transition metal nanoparticles supported La-promoted MgO as catalysts for hydrogen production via catalytic decomposition of ammonia. *J. Energy Chem.* **38**, 41 (2019).

30. Duan, X. *et al.* Tuning the size and shape of Fe nanoparticles on carbon nanofibers for catalytic ammonia decomposition. *Appl. Catal. B Environ.* **101**, 189–196 (2011).
